# A message passing framework with multiple data integration for miRNA-disease association prediction

**DOI:** 10.1038/s41598-022-20529-5

**Published:** 2022-09-28

**Authors:** Thi Ngan Dong, Johanna Schrader, Stefanie Mücke, Megha Khosla

**Affiliations:** 1 L3S Research Center, Leibniz University of Hannover, Hannover, Germany; 2 Hannover Unified Biobank (HUB), Hannover Medical School, Hannover, Germany; 3 Delft University of Technology (TU Delft), Delft, Netherlands

**Keywords:** Bioinformatics, Software

## Abstract

Micro RNA or miRNA is a highly conserved class of non-coding RNA that plays an important role in many diseases. Identifying miRNA-disease associations can pave the way for better clinical diagnosis and finding potential drug targets. We propose a biologically-motivated data-driven approach for the miRNA-disease association prediction, which overcomes the data scarcity problem by exploiting information from multiple data sources. The key idea is to enrich the existing miRNA/disease-protein-coding gene (PCG) associations via a message passing framework, followed by the use of disease ontology information for further feature filtering. The enriched and filtered PCG associations are then used to construct the inter-connected miRNA-PCG-disease network to train a structural deep network embedding (SDNE) model. Finally, the pre-trained embeddings and the biologically relevant features from the miRNA family and disease semantic similarity are concatenated to form the pair input representations to a Random Forest classifier whose task is to predict the miRNA-disease association probabilities. We present large-scale comparative experiments, ablation, and case studies to showcase our approach’s superiority. Besides, we make the model prediction results for 1618 miRNAs and 3679 diseases, along with all related information, publicly available at http://software.mpm.leibniz-ai-lab.de/ to foster assessments and future adoption.

## Introduction

Proteins are responsible for essential biological functions inside living organisms. Disruptions in proteins’ expressions are directly associated with various disease conditions^1^. Therefore, to fully characterize diseases, it is essential to investigate the regulatory network of protein-coding genes (PCGs). Among the major regulators for PCGs is a highly conserved class of non-coding RNAs with an approximate length of 22 nucleotides. These micro RNAs (miRNAs) regulate the expression of PCGs by binding to the transcribed mRNAs of PCGs, leading to the cleavage or the destabilization of the mRNAs and repressing their translation into proteins^2^.

The binding between the miRNAs and their target mRNAs is facilitated by complementary base pairing between the so-called seed region of the miRNAs and the matching sequence in the mRNAs found most often in the 3’UTR^3^. Each miRNA can have hundreds of target mRNAs. Also, each mRNA can be regulated by more than one miRNA. Though this complicated regulatory network is yet not fully understood, it is estimated that about one-third of all PCGs is regulated by at least one miRNA^4^. These ubiquitous regulatory functions are also responsible for the multitude of cell processes influenced by miRNAs: *cell development, maturation, differentiation, and apoptosis as well as cell signaling, cellular interactions, and homeostasis*^5–8^. Consequently, the mutation of miRNAs or changes in their expression can have diverse consequences that can be hard to predict. Recent studies indicate that miRNAs could serve as potential biomarkers in certain diseases such as cancers or immune-related diseases^9–15^. Identifying potential associations between miRNAs and diseases can further help in clinical diagnosis and finding potential drug targets.

While biological experiments are usually expensive and time-consuming, computational approaches, especially data-driven machine learning (ML) approaches^16–19^, can assist wet-lab experiments by predicting a potential set of associations. Early works^20–25^ focus on learning effective miRNA/disease representations from the set of known association data. The feature extraction process usually involves the computation of hand-crafted similarities. For instance, Wang et al.^20^ propose the use of miRNA functional and disease semantic similarities. Chen et al.^26^ employ Gaussian Interaction Profile (GIP) kernel similarities^27^, which are calculated directly from the miRNA-disease association data, to construct a scoring system for miRNA disease association prediction. Some other methods which rely on pre-calculated similarities include RWRMDA^22^, NetCBI^28^, RLSMDA^29^, IMCMDA^30^, Nimgcn^31^, and VGAE-MDA^32^.

More recent approaches integrate multiple such similarity features to extract useful representations and build the learning models. HGIMDA^33^ employs a heterogeneous network from miRNA functional, disease semantic, miRNA, and disease GIP similarities to build the learning model. NNMDA^34^ proposes a weighted mechanism to combine five different miRNA similarities and two disease similarities. NCMCMDA^35^ incorporates an additional neighborhood constraint to extract the final miRNA/disease representation from the integrated input similarities. DBNMDA^36^ and SAEMDA^37^ first construct the miRNA-disease pair representation from multiple miRNA/disease pre-calculated similarities. Then the two approaches employ restricted Boltzmann machines^36^ or stacked autoencoders^37^ to learn an unsupervised low-dimensional pair representation from the constructed input. EDTMDA^38^ utilizes multiple decision trees with different feature extraction strategies for effective miRNA-disease association prediction. Other similar models include MSFSP^39^, the model by Wei et al.^40^, LMTRDA^41^, MDA-SKF^42^, and SCMFMDA^43^.

Besides the data leakage problem, as already discussed in our previous work^44^, similarity-based techniques are biased toward the well-studied miRNAs and diseases^18^. Ultimately, the input features are derived from some hard-coded heuristics and assumptions, which might work effectively on the observed association set but usually do not generalize well to unseen miRNAs or diseases^18,20^. Moreover, the hard-coded heuristics cannot fully exploit the potential of the available information, for example, with respect to the association patterns or the motif/frequent subgraphs inside the miRNA-disease bipartite graph constructed from the known association set. For an in-depth review of previous works on miRNA-disease association prediction, we refer the reader to Chen et al.^45^.

Graph representation learning techniques acquired state-of-the-art performance on several machine learning problems^46,47^. They have already been applied for the miRNA-disease association prediction problem by recent works^16,22,24,25,34,48^. Chen et al.^22^ employ random walk with restart over the miRNA functional similarity network to extract useful representations that are later used as features for the prediction model. Xuan et al.^49^ exploit the *k* nearest neighbors information. Li et al.^50^ use Deepwalk to learn miRNA and disease representations for the downstream prediction task. Chen et al.^51^ utilize global network similarity. MMGCN^52^ employs a multi-view multichannel attention graph convolutional network approach. Yu et al.^53^ develops MDPBMP—a meta-path-based model over the miRNA-disease-gene heterogeneous network.

Nevertheless, a majority of the proposed models operate on the similarity network(s) constructed from hand-crafted similarity measures instead of directly learning from the raw miRNA-disease association data. Therefore, they cannot fully exploit the existing information, especially the structure patterns inside the raw association bipartite graph. A recent work^19^ proposes the use of a structural deep network embedding (SDNE) model to mine the network information directly from the miRNA-disease association graph. Nonetheless, new miRNAs or new diseases appear as isolated nodes for which SDNE cannot learn any useful representations. Therefore, the existing models still have limited prediction capability for new miRNAs or new diseases.

Other works focus more on information integration to overcome the data scarcity problem. NEMII^19^ adds miRNA family and disease semantic similarities to enrich the miRNA-disease pair representations. MMGCN^52^ proposes a multi-attention mechanism to combine multiple similarity-based measures. NNMDA^34^ employs a heterogeneous network that is constructed over five different miRNA similarities and two disease similarities for feature learning and association prediction. Ji et al.^48^ incorporate information from multiple domains, for example, miRNA-lncRNA and miRNA-PCG interactions, miRNA-drug associations, disease-lncRNA, disease-PCG associations, and disease-drug associations, to build a heterogeneous information network for feature extraction. Though promising, with respect to the added side information, current works either employ the whole raw dataset(s) or apply naive filtering steps based on the association confidence score deposited in the databases. Such naive filtering does not ensure the quality of the integrated data. Subsequently, the quality of the trained model suffers.

To this end, we propose a biologically-motivated data-driven approach that aims to counter the above challenges by jointly learning from multiple data sources. We refer to our approach as MPM. A crucial design decision of our approach includes modeling the biological relevance of miRNAs for a particular disease via the associated PCGs. We model each miRNA or disease as a directed network built from the miRNA-PCG, disease-PCG associations, and PCG-PCG functional interactions. MPM employs a message passing framework operating over the constructed networks to enrich the existing data with potential missing links or indirect connections.

To overcome the noisy data problem, we employ a *feature selection* strategy with a side-supervised task generated from the well-annotated MESH ontology^54^. Feature selection at this stage allows us to reduce the tens of thousands of associated PCGs to only the one hundred most important PCGs. This enables us to control the quality and the quantity of the added PCG-related information without introducing any additional parameters. This is extremely important, especially in the context of learning from scarce data when over-parameterized models can easily overfit.

Next, we encapsulate the enriched and filtered PCG connections into the existing miRNA-disease bipartite network to overcome the isolated nodes problem in existing works. Since PCGs are important connections between miRNA and diseases^1^, the patterns learned from the miRNA-PCG-disease interconnected networks should be a rich source of information for the miRNA-disease association prediction problem. At the same time, the newly introduced heterogeneous network will include biological connections between new miRNAs or new diseases and their associated PCGs. The learning signals will thus transfer from known miRNAs or known diseases to the new miRNAs or new diseases via the PCGs. We employ the SDNE model to extract the patterns (or pre-trained embeddings) from the constructed heterogeneous network. Besides the structural features, the final miRNA-disease pair representation is further augmented with information from the miRNA family and disease semantic similarity and then fed as input to a Random Forest classifier to perform the association prediction task.

In summary, we propose flexible information integration mechanisms at different stages of the model building process to overcome the data scarcity problem. In addition to fusing multiple knowledge sources, we propose a parameter-free mechanism to enrich and control the quality and quantity of the added data. Experimental results on 21 large independent test sets indicate that our proposed model significantly outperforms all benchmarked models in both (i) the transductive setting where we test each model’s performance on the set of partially observed miRNAs and diseases, and (ii) the inductive setting where we test the models’ performance on the set of completely new miRNAs and diseases. The three case studies’ results indicate that our approach generates reasonable predictions, even for diseases with little known knowledge. The ablation studies’ results also support our design choices for the model architecture.

We share all the code, pre-processed, and standardized data at https://git.l3s.uni-hannover.de/dong/mpm. In addition, we make the predicted association probabilities (confidence scores) for all 1618 miRNAs and 3,679 diseases publicly available at http://software.mpm.leibniz-ai-lab.de/. To enable a smooth and comprehensive analysis, we also integrate the miRNA and disease pathway and functional enrichment analysis results into the website. Section 2.6 and Section 3 in the Supplementary File provide more details regarding our website and the integrated information sources.

## Results

### Compared models

We compare our model with six recently proposed methods: (i) Epmda^16^, Dbmda^17^, and Nimgcn^31^, which utilize hand-crafted features derived from known miRNA-disease associations, (ii) MuCoMiD^18^ and DimiG 2.0^55^, which use graph convolution networks (GCNs) for feature extraction from various interaction networks (iii) NEMII^19^ which employs hand-crafted features as well as the latent features extracted using a graph embedding method. As an ablation study, we compare MPM with four of its simpler variants as summarized in Table 5. A detailed description of the compared models is provided in Section 1 in the Supplementary File. Details on hyperparameter settings and implementation for all models are provided in Section 2.6 in the Supplementary File.

### Evaluation setup

#### The testing and evaluation data setup

We first construct the Hmdd2 and Hmdd3 datasets from the HMDD v2.0^56^ and HMDD v3.0^57^ databases. While the K-fold cross-validation (K-fold CV) technique is widely used among existing works, it is insufficient to evaluate the models’ performance on completely new diseases, given the small size of the association datasets. Therefore, besides 5-fold CV evaluation on the Hmdd2 and Hmdd3 datasets, we here propose and employ two realistic testing setups: *transductive* and *inductive* to evaluate and compare models. The transductive testing setup aims at evaluating different models’ performances on a larger, independent test set which contains the newly discovered associations between the miRNAs and diseases that have already been observed with some previously known associations during the training phase. In this setup, we train each model on the Hmdd2 dataset and test it on the Held-out1 test set. Held-out1 contains only associations corresponding to the miRNAs and diseases that are observed in the Hmdd2 dataset. However, the known associations in Held-out1 do not appear in the training set Hmdd2. The inductive testing setup aims at evaluating models’ performance on completely new diseases and new miRNAs. In this setup, we conduct large-scale experiments on the 20 independent test sets to test each model’s performance on (i) a dataset with many new miRNAs (the Novel-miRNA test set), (ii) 18 complete test sets for new diseases, and (iii) a dataset with many new miRNAs and new diseases (the Held-out2 test set). For the evaluation with the Novel-miRNA and Held-out2 test sets, we train the benchmarked models with the Hmdd2 dataset. For the evaluation related to 18 new diseases, we train all models with all available association data for any disease other than the ones in the test sets. Details about the data sources, data pre-processing, and how we generate the training and testing data in both testing setups are presented in Section 2 in the Supplementary File. All datasets’ statistics are presented in Table 1 and Table 3 in the Supplementary File.

**Table 1:** The association data statistics where $|n_{md}|$, $|n_{m}|$, $|n_{d}|$ refer to the number of associations, miRNAs and diseases respectively.

Dataset	$|n_{md}|$	$|n_{m}|$	$|n_{d}|$
Hmdd2	4592	442	309
Hmdd3	10,494	742	545
Hmdd2 $\cup$ Hmdd3	10,980	742	591
Held-out1	4311	382	226
Held-out2	6388	697	509
Novel-miRNA	4734	638	227

#### Evaluation metrics

For non-parametric metrics, we report the Area under the Receiver Operating Characteristic (AUC), the Average Precision (AP) (which summarizes the Precision-Recall curve). We report the AP instead of the AUPR score because AP provides a better performance estimate than the AUPR, as discussed in our previous work in^44^. AP is calculated as the discrete sum of the changes in the recall at different thresholds instead of linear interpolation as that of AUPR, which can be too optimistic in cases where the number of thresholds (unique prediction values) is limited^58,59^. For threshold-based metrics, we report the Sensitivity (or Recall, referred to as SN), Specificity (SP), Accuracy (ACC), Precision (Pre), F1, and Matthews correlation coefficient (MCC) scores. Besides, for the new disease test sets, we also report the number of correctly predicted miRNA-disease associations among the top 100 highest predicted scores (denoted as Top100) generated by the benchmarked models. For all tables, bold font is used to highlight the best scores.

### MPM vs. existing works (SOTA)

Tables 2 and 3 present the average performance scores for all benchmarked models on our 21 large test sets in the transductive and inductive testing setups. Table 5 in the supplementary file provides detailed results with all reported metrics for the benchmarked models on the 18 test sets for new diseases. In Table 2, we report the average AP and AUC scores corresponding to different positive:negative testing sample rates. We do not have the results for Epmda on the 18 test sets for new diseases because all pairs’ representations are zeros since new diseases appear as isolated nodes in the network for the topology-based feature extraction. Table 4 shows the results corresponding to the 5-fold CV results on the Hmdd2 and Hmdd3 datasets. For each dataset, we randomly split the data according to 5 different random seeds and report the average performance.

**Table 2:** Results for all models on the three large independent test sets.

Method	Held-out1						Novel-miRNA						Held-out2					
	$nr=1$		$nr=5$		$nr=10$		$nr=1$		$nr=5$		$nr=10$		$nr=1$		$nr=5$		$nr=10$	
	AUC	AP	AUC	AP	AUC	AP	AUC	AP	AUC	AP	AUC	AP	AUC	AP	AUC	AP	AUC	AP
Nimgcn	0.542	0.554	0.541	0.207	0.542	0.118	0.532	0.549	0.53	0.202	0.53	0.115	0.513	0.517	0.513	0.176	0.512	0.097
Dbmda	0.657	0.622	0.656	0.256	0.656	0.149	0.644	0.621	0.645	0.261	0.645	0.153	0.638	0.617	0.638	0.257	0.638	0.15
Epmda	0.698	0.624	0.698	0.256	0.698	0.148	0.716	0.643	0.718	0.281	0.719	0.167	0.704	0.648	0.703	0.291	0.704	0.176
NEMII	0.838	0.831	0.838	0.542	0.838	0.395	0.865	0.857	0.866	0.597	0.866	0.452	0.859	0.853	0.859	0.581	0.858	0.435
MuCoMiD	0.832	0.826	0.832	0.534	0.832	0.385	0.827	0.819	0.827	0.519	0.827	0.37	0.811	0.812	0.812	0.514	0.811	0.368
DimiG 2.0	0.499	0.5	0.499	0.167	0.499	0.091	0.499	0.5	0.499	0.167	0.499	0.091	0.499	0.5	0.499	0.167	0.499	0.091
SOTA Improvement	*1.2%*	*1.6%*	*1.2%*	*5.7%*	*1.2%*	*8.6%*	*0.5%*	*1.1%*	*0.5%*	*3.4%*	*0.5%*	*6.0%*	*0.5%*	*1.4%*	*0.5%*	*6.9%*	*0.5%*	*11.5%*
MPM-no-MP	0.846	0.84	0.846	0.564	0.847	0.418	0.866	0.859	0.866	0.602	0.867	0.46	0.859	0.86	0.859	0.607	0.859	0.468
MPM-no-FS	0.814	0.809	0.814	0.503	0.814	0.357	0.823	0.818	0.823	0.519	0.823	0.373	0.814	0.819	0.814	0.533	0.814	0.391
MPM-no-MPFS	0.824	0.816	0.824	0.516	0.824	0.369	0.836	0.828	0.836	0.538	0.836	0.391	0.831	0.832	0.831	0.554	0.831	0.411
MPM-no-SDNE	0.837	0.83	0.837	0.546	0.837	0.401	0.842	0.834	0.842	0.552	0.843	0.408	0.846	0.847	0.846	0.581	0.846	0.439
MPM (ours)	**0.848**	**0.844**	**0.848**	**0.573**	**0.848**	**0.429**	**0.869**	**0.866**	**0.87**	**0.62**	**0.87**	**0.479**	**0.863**	**0.865**	**0.863**	**0.621**	**0.862**	**0.485**

The percentage of improvement over the state-of-the-art models are in italics.

$nr=1$, $nr=5$, $nr=10$ indicate that we test all models with the positive:negative rates of 1:1, 1:5, 1:10, respectively. Bold font is used to highlight the best scores.

**Table 3:** The AP scores corresponding to the 18 complete test sets for new diseases average over 20 experimental runs.

Disease	MPM	Nimgcn	Dbmda	NEMII	MuCoMiD	DimiG 2.0	MPM-no	MPM-no	MPM-no	MPM-no
							-MP	-FS	-MPFS	-SDNE
D001749	**0.785**	0.089	0.340	0.77	0.446	0.103	0.77	0.567	0.589	0.58
D001943	0.824	0.160	0.507	**0.827**	0.414	0.205	0.811	0.679	0.693	0.654
D002289	**0.802**	0.108	0.303	0.800	0.278	0.132	0.795	0.662	0.678	0.589
D002292	**0.684**	0.082	0.238	0.653	0.285	0.087	0.67	0.51	0.525	0.531
D002294	**0.669**	0.186	0.241	0.608	0.384	0.064	0.646	0.529	0.531	0.493
D003110	**0.659**	0.069	0.242	0.600	0.271	0.078	0.619	0.487	0.54	0.515
D005909	**0.736**	0.123	0.369	0.712	0.418	0.109	0.726	0.597	0.63	0.523
D005910	0.759	0.112	0.246	0.731	0.409	0.117	**0.767**	0.642	0.66	0.626
D006333	0.669	0.180	0.300	0.651	0.395	0.088	**0.671**	0.578	0.602	0.566
D008175	**0.764**	0.115	0.437	0.749	0.375	0.138	0.751	0.615	0.62	0.611
D008545	**0.724**	0.108	0.355	0.706	0.365	0.117	0.715	0.58	0.598	0.558
D010051	**0.792**	0.114	0.400	0.760	0.388	0.118	0.782	0.505	0.654	0.579
D010190	0.749	0.088	0.366	0.744	0.373	0.098	**0.761**	0.589	0.622	0.598
D011471	0.733	0.116	0.395	0.653	0.330	0.135	**0.738**	0.618	0.633	0.569
D012516	**0.713**	0.262	0.323	0.658	0.349	0.098	0.699	0.546	0.585	0.55
D013274	**0.837**	0.132	0.503	0.835	0.249	0.161	0.811	0.657	0.693	0.643
D015179	**0.806**	0.134	0.463	0.797	0.340	0.171	0.785	0.645	0.693	0.614
D015470	**0.655**	0.158	0.259	0.625	0.290	0.069	0.653	0.509	0.513	0.497

**Table 4:** Results for 5-fold cross-validation on the Hmdd2 and Hmdd3 datasets.

Dataset	Method	AUC	AP	Sensitivity	Specificity	Accuracy	Precision	F1	MCC
HMDD2	MPM	0.89 ± 0.01	**0.9 ± 0.01**	80.7 ± 1.2	81.5 ± 1.4	81.1 ± 1.0	81.3 ± 1.2	81.0 ± 1.0	62.2 ± 2.1
	Nimgcn	0.88 ± 0.01	0.87 ± 0.01	70.2 ± 26.0	**84.2 ± 6.4**	77.2 ± 10.1	77.9 ± 17.6	71.0 ± 26.2	54.6 ± 19.9
	Dbmda	0.72 ± 0.01	0.68 ± 0.01	66.9 ± 1.6	72.4 ± 1.8	69.7 ± 1.1	70.8 ± 1.3	68.8 ± 1.1	39.4 ± 2.1
	Epmda	0.52 ± 0.02	0.61 ± 0.02	36.0 ± 48.0	64.0 ± 48.0	50.0 ± 0.0	18.0 ± 24.0	24.0 ± 32.0	0.0 ± 0.0
	NEMII	0.9 ± 0.01	**0.9 ± 0.01**	81.4 ± 1.1	81.5 ± 1.6	81.4 ± 1.0	81.5 ± 1.3	81.4 ± 0.9	62.9 ± 2.0
	MuCoMiD	**0.91 ± 0.01**	**0.9 ± 0.01**	83.0 ± 2.3	82.5 ± 2.2	**82.8 ± 1.0**	**82.7 ± 1.6**	**82.8 ± 1.0**	**65.6 ± 1.9**
	DimiG 2.0	0.5 ± 0.01	0.51 ± 0.01	**100.0 ± 0.0**	0.0 ± 0.0	50.0 ± 0.0	50.0 ± 0.0	66.7 ± 0.0	0.0 ± 0.0
HMDD3	MPM	0.91 ± 0.0	0.91 ± 0.01	83.8 ± 0.8	82.0 ± 0.9	82.9 ± 0.6	82.3 ± 0.8	83.0 ± 0.6	65.8 ± 1.2
	Nimgcn	0.89 ± 0.01	0.89 ± 0.01	84.6 ± 1.7	80.7 ± 2.1	82.7 ± 0.7	81.5 ± 1.5	83.0 ± 0.7	65.4 ± 1.4
	Dbmda	0.76 ± 0.01	0.71 ± 0.01	71.6 ± 1.2	74.4 ± 1.1	73.0 ± 0.6	73.7 ± 0.7	72.6 ± 0.7	46.1 ± 1.1
	Epmda	0.48 ± 0.01	0.59 ± 0.01	48.0 ± 50.0	52.0 ± 50.0	50.0 ± 0.0	24.0 ± 25.0	32.0 ± 33.3	0.0 ± 0.0
	NEMII	0.91 ± 0.0	0.91 ± 0.01	84.1 ± 0.7	82.0 ± 1.0	83.0 ± 0.6	82.4 ± 0.8	83.2 ± 0.6	66.1 ± 1.2
	MuCoMiD	**0.92 ± 0.0**	**0.92 ± 0.01**	85.2 ± 1.7	**84.0 ± 1.2**	**84.6 ± 0.7**	**84.2 ± 0.9**	**84.7 ± 0.8**	**69.2 ± 1.5**
	DimiG 2.0	0.5 ± 0.0	0.5 ± 0.0	**100.0 ± 0.0**	0.0 ± 0.0	50.0 ± 0.0	50.0 ± 0.0	66.7 ± 0.0	0.0 ± 0.0

In the three large independent test sets (ref. Table 2), MPM outperforms all benchmarked models (SOTA) on the Held-out1 (transductive setting), Novel-miRNA (with many new miRNAs), and Held-out2 (with new miRNAs and new diseases) test sets with a gain of up to 11.5% in AP score. The gains are more significant when more negative samples are added to the testing data. On the complete test sets for new diseases, MPM consistently acquires the highest Top100 scores in all test sets. Besides, MPM gains the highest AP scores in 17 out of 18 datasets. In the 5-fold CV evaluation setup, MuCoMiD gains the highest performance in most reported metrics. MPM closely follows NEMII with slightly worse performance. Nonetheless, compared to the best-performing model (MuCoMiD), MPM attains an equal AP score in the Hmdd2 dataset and a 0.01 lower AP score in the Hmdd3 dataset.

In both transductive and inductive testing setups, we observe similar trends with large performance gaps among the state-of-the-art methods. In the three large independent test sets (Held-out1, Novel-miRNA, Held-out2), DimiG 2.0 performs the worst, followed by Nimgcn, then Dbmda, Epmda, MuCoMiD, and then NEMII. In the 18 complete test sets for new diseases, regarding the AP scores, the order is slightly changed to Nimgcn, followed by DimiG 2.0, then Dbmda, MuCoMiD, and then NEMII. DimiG 2.0 is a recently proposed model that formulates the miRNA-disease association prediction problem as a semi-supervised node classification task with diseases as labels. The model can integrate information from four additional knowledge sources (miRNA-PCG, disease-PCG associations, PCG-PCG interactions, and disease ontology) but only performs training using the known disease-PCG association set. Though DimiG 2.0 can generate predictions for new miRNAs and new diseases, the large and sparse label set and the weak training signals lead to its limited predictive performance. With all AUC scores close to 0.5, the model does not perform better than a random guess.

Nimgcn performs the worst compared to other supervised baselines because it only relies on the miRNA functional and disease semantic similarities to construct the networks for the feature learning. The miRNA functional similarity is heavily biased toward well-known diseases and cannot generalize well to new diseases^20^. Also, new miRNAs appear as isolated nodes in the network and will get completely random representations. Therefore, Nimgcn ’s prediction capability is limited for the little-known or completely new miRNAs or diseases.

Regarding the input sources, Dbmda improves over Nimgcn by integrating another biologically-related information source: the miRNA sequence similarity. Dbmda gains significantly better performance than Nimgcn but is still much lower than MuCoMiD, NEMII, and MPM in most test sets, suggesting that the miRNA sequence similarity does bring additional benefit, but the gains are not too significant.

Epmda proposes a topologically related feature extraction technique for miRNA-disease pair representation. Unlike most existing works, which focus on learning effective representations for miRNAs and diseases separately, Epmda learns the miRNA-disease pair representation directly as a property of the miRNA-disease heterogeneous network constructed from the miRNA and disease Gaussian Interaction Profile kernel similarities and the miRNA-disease known associations. Even though Epmda does not employ any additional information sources, its performance is still better than Nimgcn and Dbmda. This suggests that learning the pair representation directly from the heterogeneous network with raw miRNA-disease associations is a fruitful direction. Nonetheless, the edge perturbation score has at least $O(n^3)$ time complexity and cannot scale well to a large network^44^. Besides, fine-tuning the network cycle length parameter is not a trivial task^44^.

MuCoMiD proposes a multitask learning model that integrates five additional information sources to overcome the data scarcity problem. Though promising, the model applies hard-threshold filtering to filter out redundant information in the additional information sources. The results reported in Tables 2 and 3 correspond to MuCoMiD ’s performance without the filtering step (since not all of our data have the interaction/association confidence scores available). The thresholds need to be fine-tuned for each dataset separately. For that reason, it requires considerable time and effort for parameter fine-tuning in order to employ MuCoMiD for a completely new dataset. This points to an important aspect of information integration which focuses on effectively controlling/managing the quality and quantity of the added knowledge sources. Nonetheless, MuCoMiD gains the highest performance in the 5-fold CV testing setup. Also, the method shows promising performance, which overcomes the problems associated with hand-crafted similarity-based methods in all testing setups.

NEMII learns structural embeddings directly from the miRNA-disease bipartite network constructed from the known miRNA-disease association data. Besides, the model is further informed by information from the miRNA family and disease semantic similarity. Though new miRNAs and new diseases get completely random representation from the structural embedding learning module, NEMII ’s performance on the 20 inductive testing datasets is still one of the highest, thanks to the biological information from the miRNA family and disease semantic similarity features. Overall, the effective feature extraction strategy, combined with the domain knowledge from the added side information sources, helped NEMII gain the highest performance scores among state-of-the-art methods on most testing datasets. These results support the exploitation of structural information from the miRNA-disease association data and the importance of information integration.

MPM improves over state-of-the-art methods with a parameter-free yet effective mechanism to control the quality and quantity of the added information sources. At the same time, it addresses the existing limitation in the NEMII model by integrating additional biological relations to the new miRNAs and new diseases. The learned signals from the well-studied miRNAs/diseases will be transferred to the diseases (with only scarce knowledge) via their associated PCGs. These improvements help MPM gain state-of-the-art performance on 20 out of the 21 independent test sets in both transductive and inductive testing setups with a gain of up to 11.5% in AP score.

### Ablation studies

Here, we compare MPM with four of its simpler variants as summarized in Table 5.

**Table 5:** Simpler variants of MPM where ‘✓’ and ‘×’ denote the existence and non-existence of the corresponding components/modules.

Model	Message Passing	Feature Selection	SDNE	Random Forest classifier	PCG associations
MPM-no-MP	×	✓	✓	✓	✓
MPM-no-FS	✓	×	✓	✓	✓
MPM-no-SDNE	✓	✓	×	✓	✓
NEMII^19^	×	×	✓	✓	×
MPM-no-MPFS	×	×	✓	✓	✓

MPM-no-MP is a variant of MPM without the message passing layer that takes the raw miRNA-PCG and disease-PCG associations as input to the feature selection and structural embedding learning modules. Similarly, MPM-no-FS is a variant of MPM without the feature selection module. The structural embedding learning module encapsulates all enriched miRNA-PCG and disease-PCG associations output from the message passing layer into its heterogeneous network for learning node embeddings. MPM-no-MPFS is a variant of MPM without the message passing and the feature selection modules. The heterogeneous network input to SDNE simply integrate all raw miRNA-PCG, disease-PCG associations retrieved from miRTarBase^60^ and DisGeNET^61^. MPM-no-SDNE is a variant of MPM in which there is no structural embedding learning. Instead, the pair representation for a particular miRNA-disease pair is the concatenation of the enriched and filtered miRNA-PCG, disease-PCG associations, miRNA family, and disease semantic similarity features.

Table 2 presents the results for MPM and its variants on three large independent test sets. Table 3 reports the results for the 18 inductive testing datasets for new diseases. We observe that MPM supersedes all of its simpler variants on the transductive testing set (Held-out1), two inductive testing sets with many new miRNAs (Novel-miRNA and Held-out2), and 15 out of 18 complete test sets for new diseases. The gains are the most significant on the three independent test sets (c.f. Table 2), especially when more negative testing samples are added. These results support the contribution of each added component. At the same time, they validate our choice of architecture.

Besides, among the simpler variants, we observe a considerable performance drop on the variants without the feature selection modules (MPM-no-FS and MPM-no-MPFS) or on the MPM-no-SDNE model. Without the feature selection module, the network employed for the embeddings generation contains too many PCG association connections. As biological data usually contains many false positives, adding all PCG associations introduces additional noise and redundancy. Similarly, without the structural embeddings (MPM-no-SDNE), MPM only relies on the associated PCGs, miRNA, and disease semantic similarity features to generate predictions without the information about the miRNA/disease interaction patterns. The drop in performance observed in MPM ’s simpler variants further emphasizes the importance of our feature selection module for information filtering as well as the SDNE module for feature extraction from the raw association structural patterns.

An ablation study comparing Random Forest with six other binary classifiers is presented in Section 3.1 and Table 4 in the Supplementary File.

### Case studies

Let **H** = Hmdd2 ∪ Hmdd3 denote the set of all known associations retrieved from the HMDD databases. We here present three case studies to showcase the application of MPM in realistic scenarios.

#### MPM for a disease with scarce knowledge

*Down syndrome* or Trisomy 21 is a condition in which a child is born with an extra copy of their 21st chromosome^62^. *Down Syndrome*’s patients usually suffer from mild-to-moderate learning disabilities^62^. According to the data deposited in the HMDD 2.0 and HMDD 3.0 databases and two recent works^63,64^, there are only 10 miRNAs known to be associated with the disease of our interest. We assume that *Down Syndrome* is a completely new disease and take similar steps as those presented in Section 2.4.2 in the Supplementary File to construct the training and testing data. In short, our training data consists of all known associations in $\mathbf {H}$ for all diseases other than the *Down Syndrome*. We test MPM on the complete test set consisting of all possible combinations between the *Down Syndrome* and 1618 miRNAs.

**Table 6:** MPM ’s average prediction scores for *Down Syndrome* and all 1618 miRNAs.

Rank	miRNA	Pred.	Rank	miRNA	Pred.
	.		*82*	*hsa-mir-125b-2*	*0.579253110400618*
*2*	*hsa-mir-155*	*0.963881105523116*		.	
*3*	*hsa-mir-146a*	*0.934014942433006*	*105*	*hsa-mir-99a*	*0.482246263067031*
*4*	*hsa-mir-16-1*	*0.895608127697913*		.	
	.		*140*	*hsa-mir-1246*	*0.404202397336283*
*33*	*hsa-mir-27b*	*0.689694528927961*		.	
	.		*261*	*hsa-let-7c*	*0.244887327696169*
*38*	*hsa-mir-27a*	*0.671913693062923*		.	
	.		*1576*	*hsa-mir-802*	*0.130087980984639*

The associated miRNAs are marked as italics. The model training data does not contain the association data for *Down Syndrome*.

##### How effective is MPM in restricting and prioritizing the search space for the potentially associated miRNAs?

Table 6 presents the average predictions made by MPM after 20 experimental runs. Though we perform the search on a complete test set of 1618 testing samples, 3 known-to-associate miRNAs (marked as italics in Table 6 already appear in the top 4 highest predicted results. The other associated miRNAs appear at 33th, 38th, 82th, 105th, 140th, 261th, and 1576th positions in the prediction list. With 3 appearing in the top 4 and 5 out of 10 known associations appearing in the top 38 of the generated prediction results, our method would significantly help restrict and prioritize the search space for wet-lab experiments.

##### How effective is MPM with some added domain knowledge?

Since *Down Syndrome* relates to a redundant chromosome 21 copy, we retrieve the miRNA location information from miRTarBase^60^ and present MPM ’s predicted results for all miRNAs located on chromosome 21 in Table 7. Italics is used to mark the associated miRNAs.

**Table 7:** MPM ’s prediction results for *Down Syndrome* and the miRNAs that are located on chromosome 21.

Rank	miRNA	Pred.	Rank	miRNA	Ped.
*1*	*hsa-mir-155*	*0.963881105523116*	11	hsa-mir-4760	0.172962854437391
*2*	*hsa-mir-125b-2*	*0.579253110400618*	12	hsa-mir-5692b	0.168364046134056
*3*	*hsa-mir-99a*	*0.482246263067031*	13	hsa-mir-6508	0.163143029370321
*4*	*hsa-let-7c*	*0.244887327696169*	14	hsa-mir-6070	0.16232917173827
5	hsa-mir-548x	0.239129159103197	15	hsa-mir-6815	0.159395572782035
6	hsa-mir-3648-1	0.206785057828119	16	hsa-mir-8069-1	0.155993241075239
7	hsa-mir-4759	0.200771150543586	17	hsa-mir-6724-1	0.153456269809843
8	hsa-mir-3197	0.19795748172893	18	hsa-mir-6501	0.152740622433185
9	hsa-mir-6130	0.194382789321313	19	hsa-mir-6814	0.145666592873055
10	hsa-mir-4327	0.176297567535453	*20*	*hsa-mir-802*	*0.130087980984639*

Italics is used to highlight the associated miRNAs. The model training data does not contain the association data for *Down Syndrome*.

By restricting the miRNA search space, we have much more promising prediction results, with 4 out of 5 associated miRNAs appearing at the top of the list. Adding more related domain information like chromosomal location, tissue expression profiles, etc., thus helps in restricting the miRNA search space to obtain more meaningful prediction results. Nonetheless, we release predicted association probabilities for all 1618 miRNAs to encourage field experts’ assessments as well as to enable them to perform customized subset selection without the need to retrain/rerun the model.

#### MPM for a disease with many false positives

*Parkinson* disease (PD) is the second most common neurodegenerative disease worldwide^65^. Existing human association studies for the *Parkinson* disease resulted in inconsistent findings with many “false positives” as reported in^66^. In this case study, we take a closer look at the generated predictions from MPM for the *Parkinson* disease. We train MPM with all the available data in $\mathbf {H}$. More specifically, besides the data for other diseases, the training data contains 61 known associations for *Parkinson*. Among those, there are 8 true positives (those that are confirmed as positives in^66^) and 26 false positives^66^ (those that are marked as positive in $\mathbf {H}$ but are confirmed as negative in^66^).

**Table 8:** The predicted association probabilities for the *true positive* (marked as italics) and *true negative* miRNAs^66^ corresponding to the *Parkinson* disease.

Rank	miRNA	Pred.	Rank	miRNA	Pred.	Rank	miRNA	Pred.	Rank	miRNA	Pred.	Rank	miRNA	Pred.
1	hsa-mir-7-1	0.99	23	hsa-mir-127	0.96	45	hsa-mir-99a	0.92	67	hsa-mir-25	0.85	89	hsa-mir-149	0.62
2	hsa-mir-30d	0.99	24	hsa-mir-145	0.96	46	hsa-mir-19a	0.92	68	hsa-mir-23a	0.85	90	hsa-mir-1264	0.62
*3*	*hsa-mir-19b-1*	0.99	25	hsa-mir-195	0.96	*47*	*hsa-mir-29c*	0.92	69	hsa-mir-191	0.85	91	hsa-mir-744	0.61
*4*	*hsa-mir-146a*	0.99	*26*	*hsa-mir-497*	0.96	48	hsa-mir-1301	0.91	70	hsa-mir-140	0.84	92	hsa-mir-301b	0.6
5	hsa-mir-335	0.99	27	hsa-mir-338	0.96	49	hsa-mir-30b	0.91	71	hsa-mir-136	0.83	93	hsa-mir-154	0.59
*6*	*hsa-mir-193a*	0.99	28	hsa-mir-222	0.96	50	hsa-mir-152	0.9	72	hsa-mir-16-2	0.82	94	hsa-mir-184	0.55
*7*	*hsa-mir-214*	0.98	*29*	*hsa-mir-221*	0.96	51	hsa-mir-125b-2	0.9	73	hsa-mir-98	0.82	95	hsa-mir-223	0.54
*8*	*hsa-mir-141*	0.98	30	hsa-mir-22	0.96	52	hsa-mir-125a	0.9	74	hsa-mir-27b	0.81	96	hsa-mir-532	0.49
9	hsa-mir-151a	0.98	31	hsa-mir-299	0.96	53	hsa-mir-137	0.9	75	hsa-mir-345	0.81	97	hsa-mir-1296	0.48
10	hsa-mir-126	0.98	32	hsa-mir-424	0.95	54	hsa-mir-204	0.89	76	hsa-mir-142	0.8	98	hsa-mir-873	0.44
11	hsa-mir-7-2	0.98	33	hsa-mir-21	0.95	55	hsa-mir-224	0.89	77	hsa-mir-708	0.8	99	hsa-mir-125b-1	0.42
12	hsa-mir-146b	0.98	34	hsa-mir-17	0.95	56	hsa-mir-148b	0.89	78	hsa-mir-1249	0.78	100	hsa-mir-1298	0.35
13	hsa-mir-29b-2	0.98	35	hsa-mir-148a	0.94	57	hsa-mir-409	0.89	79	hsa-mir-190a	0.78	101	hsa-mir-939	0.34
14	hsa-mir-30a	0.98	36	hsa-mir-143	0.94	58	hsa-mir-504	0.89	80	hsa-mir-129-1	0.77	102	hsa-mir-488	0.29
15	hsa-mir-199b	0.98	37	hsa-mir-28	0.94	59	hsa-mir-186	0.89	81	hsa-mir-331	0.76	103	hsa-mir-330	0.24
16	hsa-mir-34c	0.98	38	hsa-mir-425	0.93	60	hsa-mir-448	0.88	82	hsa-mir-181c	0.75	104	hsa-mir-192	0.2
*17*	*hsa-mir-132*	0.98	39	hsa-mir-10b	0.93	61	hsa-mir-769	0.87	83	hsa-mir-150	0.73	105	hsa-mir-626	0.19
*18*	*hsa-mir-451a*	0.97	*40*	*hsa-mir-29a*	0.93	62	hsa-mir-1248	0.87	84	hsa-mir-489	0.72	106	hsa-mir-26b	0.16
*19*	*hsa-mir-133b*	0.97	41	hsa-mir-99b	0.93	63	hsa-mir-92a-2	0.87	85	hsa-mir-505	0.68	107	hsa-mir-577	0.16
20	hsa-mir-10a	0.97	42	hsa-mir-543	0.93	64	hsa-mir-328	0.86	86	hsa-mir-203a	0.67	108	hsa-mir-654	0.15
21	hsa-mir-16-1	0.97	43	hsa-mir-34b	0.93	65	hsa-mir-92a-1	0.86	87	hsa-mir-454	0.65	109	hsa-mir-378a	0.15
22	hsa-mir-30c-2	0.97	44	hsa-mir-431	0.92	66	hsa-mir-20a	0.85	88	hsa-mir-130a	0.64	110	hsa-mir-501	0.12

We present the predicted association probabilities for all 12 *true positive* and 98 *true negative* miRNAs retrieved from the meta analysis^66^ corresponding to the *Parkinson* disease in Table 8. Though the training data contains more than three folds of the false-positive associations (26 false positives vs. 8 true positives), we observe that all 12 true positives reported in^66^ could be found in the top 50 predictions. Among those, 5 out of 12 appear in the top 8, while 8 out of 12 show up in the top 19 predictions. These results support that MPM acquires good performance in differentiating between the true positive and true negative miRNAs even with the noisy training data.

#### Survival analysis for Precursor B-cell lymphoblastic leukemia

Precursor B-cell lymphoblastic leukemia (PBLL) is the most common type of Acute lymphoblastic leukemia that is characterized by a high number of B-cell lymphoblasts found in blood and bone marrow. According to the data deposited in the HMDD databases, there are 7 miRNAs known to be associated with PBLL. In this case study, we perform survival analysis on PBLL patients’ data.

##### miRNA expression and survival outcome

We download the miRNA expression and survival information for PBLL patients from TCGA Genomic Data Commons (GDC)^67^ using the GDC Data Transfer Tool^68^. As a pre-processing step, we remove the patients without survival information and retain only the records that have the *Sample Type* as *Primary Tumor*. For the patients that have only one sample, the miRNA expression values are taken as the read per million values. For each patient with more than one sample, each miRNA expression value is calculated as the average of all the available reads per million values. The final pre-processed data contains the miRNA expression profiles and survival outcomes for 167 PBLL patients. For each miRNA, we use StepMiner^69^ to compute a threshold that can robustly differentiate between the high and low expression levels. The computed thresholds are used to discretize the data so that the miRNA continuous expression values can be divided into high, intermediate, and low expression classes. We use the log-rank test^70–72^ to assess the statistical significance of the survival difference between the high and low expression classes. The Kaplan-Meier analysis and log-rank test are performed using the *lifelines*^73^ package.

##### MPM prediction

We train MPM with all known associations deposited in the HMDD databases for all diseases other than PBLL and generate MPM ’s prediction scores for all 1618 miRNAs.

**Figure 1:**
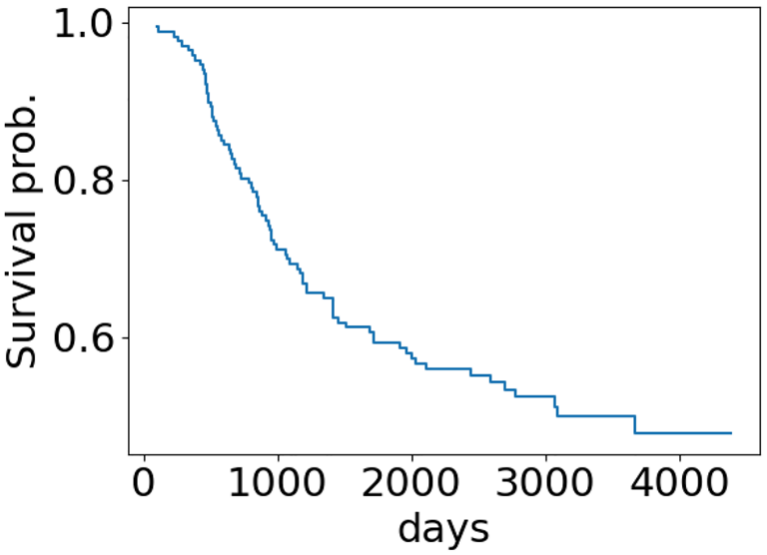
The Kaplan survival curve of PBLL patients.

##### Results

**Figure 2:**
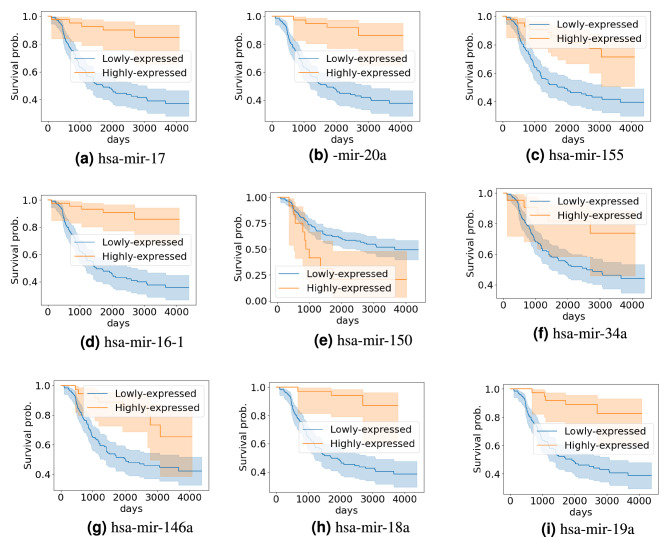
Kaplan–Meyer survival curves of PBLL patients stratified by the top miRNAs with the top highest prediction scores.

The Kaplan-Meier survival curve for PBLL patients is presented in Fig. 1. According to the log-rank test results, we identify 310 miRNAs associated with patients’ survival outcomes with a p-value $< 0.05$. We refer to this set as $\mathcal {L}$. We observe that none of the known-to-be-associated miRNAs (deposited in the HMDD databases) appear in $\mathcal {L}$. But from the top 10 miRNAs that have the highest prediction scores generated by MPM, 8 already appear in $\mathcal {L}$. Among the top 20 miRNAs that have the highest prediction scores, 13 already appear in $\mathcal {L}$. Table 9 presents the top miRNAs that have the highest prediction scores that appear in $\mathcal {L}$, along with their rank in MPM ’s prediction list. The full list of $\mathcal {L}$ and all MPM ’s prediction scores can be downloaded from https://git.l3s.uni-hannover.de/dong/mpm/-/tree/master/PBLL. Figure 2 shows the Kaplan-Meir survival curves of PBLL patients stratified by the top miRNAs that have the highest prediction scores generated by MPM. All things considered, for PBLL, MPM prediction results agree well with the survival analysis results. This further supports the applicability of MPM in identifying potential prognostic miRNAs for complex diseases.

**Table 9:** The top miRNAs with the highest prediction scores that appear in $\mathcal {L}$—the list of associated miRNAs output from the survival analysis.

Rank	miRNA	Pred.	Rank	miRNA	Pred.	Rank	miRNA	Pred.	Rank	miRNA	Pred.
2	hsa-mir-17	0.98	17	hsa-mir-145	0.93	37	hsa-mir-130a	0.84	58	hsa-mir-200c	0.75
3	hsa-mir-20a	0.98	18	hsa-mir-143	0.92	38	hsa-mir-125a	0.83	61	hsa-mir-149	0.75
4	hsa-mir-155	0.98	19	hsa-mir-26a-1	0.92	40	hsa-mir-204	0.83	62	hsa-mir-100	0.74
5	hsa-mir-16-1	0.97	23	hsa-mir-31	0.91	45	hsa-mir-122	0.81	63	hsa-mir-200b	0.74
6	hsa-mir-150	0.97	24	hsa-mir-181a-2	0.9	46	hsa-mir-25	0.81	64	hsa-mir-192	0.74
7	hsa-mir-34a	0.96	25	hsa-mir-19b-1	0.9	47	hsa-mir-15b	0.81	71	hsa-mir-16-2	0.73
9	hsa-mir-146a	0.95	27	hsa-mir-22	0.89	48	hsa-mir-148a	0.8	72	hsa-mir-98	0.73
10	hsa-mir-18a	0.95	29	hsa-mir-92a-1	0.86	51	hsa-mir-132	0.79	73	hsa-mir-107	0.72
14	hsa-mir-19a	0.94	31	hsa-mir-106b	0.85	54	hsa-mir-106a	0.78	75	hsa-mir-335	0.72
15	hsa-mir-15a	0.94	33	hsa-mir-181b-1	0.85	56	hsa-mir-378a	0.76	76	hsa-mir-26b	0.72

### An integrated, easy-to-use website for comprehensive analyses

We provide an easy-to-use website to query the predictions generated by our proposed model on 1618 miRNAs and 3679 diseases at http://software.mpm.leibniz-ai-lab.de/. It is important to note that the model is trained only from the data corresponding to only a few hundred miRNAs and a few hundred diseases. We offer a large computational prediction capability for thousands of available diseases and miRNAs through the website. All the results corresponding to the pathway and the enrichment analysis for all miRNAs and diseases are also generated and integrated to enable a comprehensive analysis by the field experts. Besides, the users can also (i) search for miRNAs in the same family or related diseases (i.e., parents/children in the disease ontology) through the provided search capabilities, (ii) analyze pathways and GO processes for an input miRNA or disease, and (iii) query the list of miRNAs or diseases associated with a particular pathway. A detailed user guide with some screenshots of the website is provided in Section 3 in the Supplementary File.

## Conclusion and future outlook

We propose a message passing framework with multiple data sources integration, MPM, for the problem of predicting miRNA-disease associations. MPM exploits information from multiple data sources to enrich and filter the raw biologically relevant features without introducing additional parameters. Besides detecting new associations of the partially observed miRNAs or diseases, MPM can successfully generate predictions for new diseases (which has no prior observed association in the training data). Our case studies further support (i) the reliability of MPM for predicting associations for diseases with scarce knowledge and (ii) its robustness in ranking the true positives higher when many false positives are present in the training data. In addition, MPM generated predictions for the PBLL disease agree quite well with the results retrieved from survival analysis on the publicly available miRNA expression data. Besides the proposed machine learning model, we also make the generated predictions more accessible to non-expert users by encapsulating all the generated and related domain information into a publicly available website. By releasing such a user-friendly interface, we aim to foster assessments and future adoption.

### Future outlook

In our opinion, the potential future directions for miRNA-disease association prediction tools include enhancement of model interpretability, input data quality, and user experience. For example, one can employ post-hoc explanation techniques^74^ to generate instance-level explanations. Nevertheless, domain expertise will be required to translate these explanations into biological rationales. In addition, one can also focus on input data or feature enhancements that include but are not limited to data filtering, additional data integration, and robust or credible negative sample selection strategies.

Works that focus on *user experience enhancement* should provide a user-friendly interface like a portable application or a publicly available website. Besides, some of the nice-to-have features of the tool would include (i) automation of data/results filtering with different filtering criteria, (ii) comparison of the generated predictions from different models, (iii) the possibility to train the model on the fly with user-customized input data, and (iv) allowing configurable model parameters. Besides, integrating more related biological information like the miRNA tissue expression profile, miRNA chromosomal location, clinical disease phenotype, etc., to support hypothesis testing or provide biological insights for a meaningful prediction subset selection would be useful but challenging to incorporate.

#### Potential applicability to miRNA-small molecule drug association prediction

Small molecule drugs are organic compounds with low molecular weights of around 900 Daltons. Small molecules form the majority of existing drugs and can be rapidly diffused across cell membranes^75^. Identification of miRNA-small molecule drug associations can help in disease therapy development. One of the first machine learning-based models for miRNA-small molecule drug association prediction is proposed by Jamal et al.^76^. The authors present a traditional machine learning approach that represents each miRNA-small molecule drug pair as a concatenated feature vector of miRNA and small molecule drug integrated similarities. The feature representations are then used as input to the Random Forest based binary classifier. More recent methods usually involve the use of graph representation learning techniques^77–84^, kernel methods^85^ and matrix factorization^86^. A recent review about miRNA-small molecule drug association can be found in^75^.

One shared characteristic of existing works is the utilization of small molecule drug and miRNA pre-calculated similarities. Though these works usually combine various similarities to mitigate bias and lack of information, they still suffer from issues related to the use of pre-calculated similarities, such as being hard to update and maintain^18^. Graph-based methods additionally encapsulate raw miRNA-small molecule drug associations in the constructed network but the number of known associations is usually too small compared to the similarity connections. This prevents the model from learning informative association patterns. Overall, it is essential to perform task specific information filtering to remove noise and balance the amount of side information added.

Our model architecture can be easily adapted for the miRNA-small molecule drug association prediction problem. The types of input information as utilized by our model are also available for this problem. Firstly, one can extract small molecule drug similarity features based on side effects^87^, functional consistency^88^, chemical structure^89^, and indication phenotype^87^. Secondly, we can retrieve small molecule drug-targeted genes from public databases like DrugBank^90^. Finally, each small molecule drug is also assigned to one or more ATC codes^91^, which incorporate information such as its anatomical distribution, therapeutic effects, and structural characteristics. Such ATC codes are also organized into a hierarchy with different levels of granularity, like the disease ontology in our case. Nevertheless, there are still some open questions and considerations regarding (i) the choice of similarity features, (ii) the biological rationale(s) for adding PCG associations as intermediate connecting points, and (iii) the most suitable supervised problem for performing feature selection (for example, should one use drug first level ATC code classification?). Answering such questions would require an in-depth understanding of the problem. Compared with the existing approaches, one advantage of our proposed model is that it offers a parameter-free information filtering mechanism to filter out redundant connections. High-quality input enables us to learn meaningful association patterns from the input network. Also, to the best of our knowledge, the SDNE method employed by MPM has never been used in existing works for miRNA-small molecule drug association prediction.

## Methods

**Figure 3:**
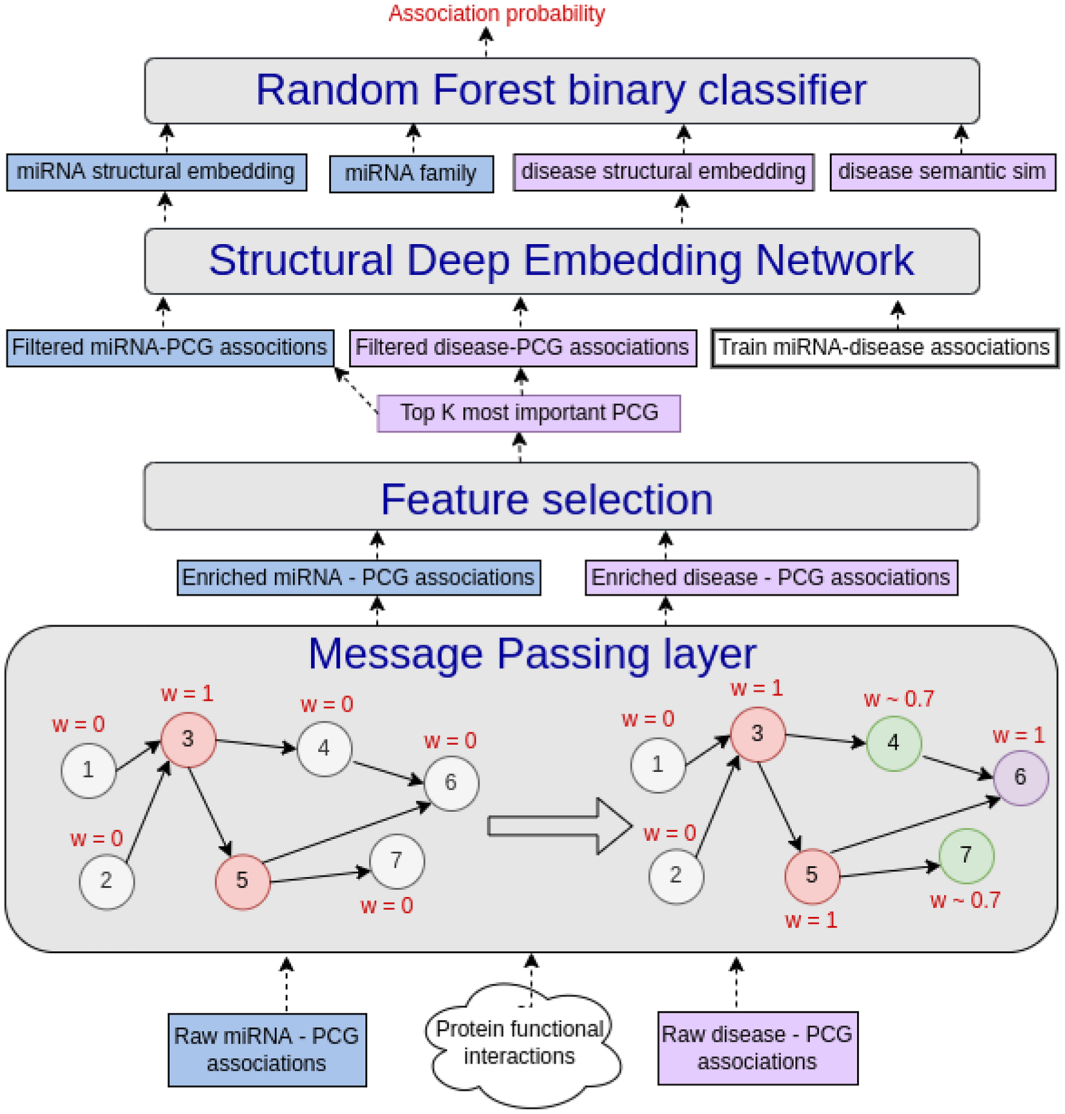
MPM’s architecture. MPM consists of a message passing layer (section “The message passing framework/module”) , a feature selection with a side supervised task (section “The feature selection module”), a Structural Deep Embedding network (section “The structural embedding learning”), and a binary classifier (section “The classification module”).

MPM treats the miRNA-disease association prediction problem as a binary classification task where the label for an input pair (*m*, *d*) is 1 if there is a known association between miRNA *m* and disease *d* and 0 otherwise. A schematic diagram of MPM with its main components is presented in Fig. 3. We use gray for the model’s components/modules, blue and violet for miRNA and disease-related components, respectively.

### The message passing framework/module

#### The data sources

Table 10 provides the statistics for our employed data sources. In the following, we describe each source in detail and present the information corresponding to how we utilize it.

**Table 10:** Statistics for the side data sources. |*E*| denotes the number of interactions/associations. $|V_m|, |V_d|,|V_p|$ represent the number of miRNAs, diseases, and PCGs, respectively.

Network	|*E*|	$|V_{m}|$	$|V_{d}|$	$|V_{p}|$
miRNA-PCG	345,357	1618	–	23,611
Disease-PCG	510,782	–	3679	23,611
Protein functional interactions	423,672			23,611

##### The protein functional interaction network

rotein coding genes (PCGs) are essential connections between miRNAs and diseases^1^. miRNAs can affect the PCG transcriptions, resulting in protein expression changes, which can then lead to diseases. Therefore, besides the knowledge about the protein-protein interactions as already exploited in^18^, the knowledge related to whether a particular protein regulates/inhibits/catalyzes/activates another protein is also very important for the miRNA-disease association prediction task. We refer to the multi-relational protein-protein interaction network, where an edge corresponds to a protein functional relation as *protein functional interaction network*.

**Figure 4:**
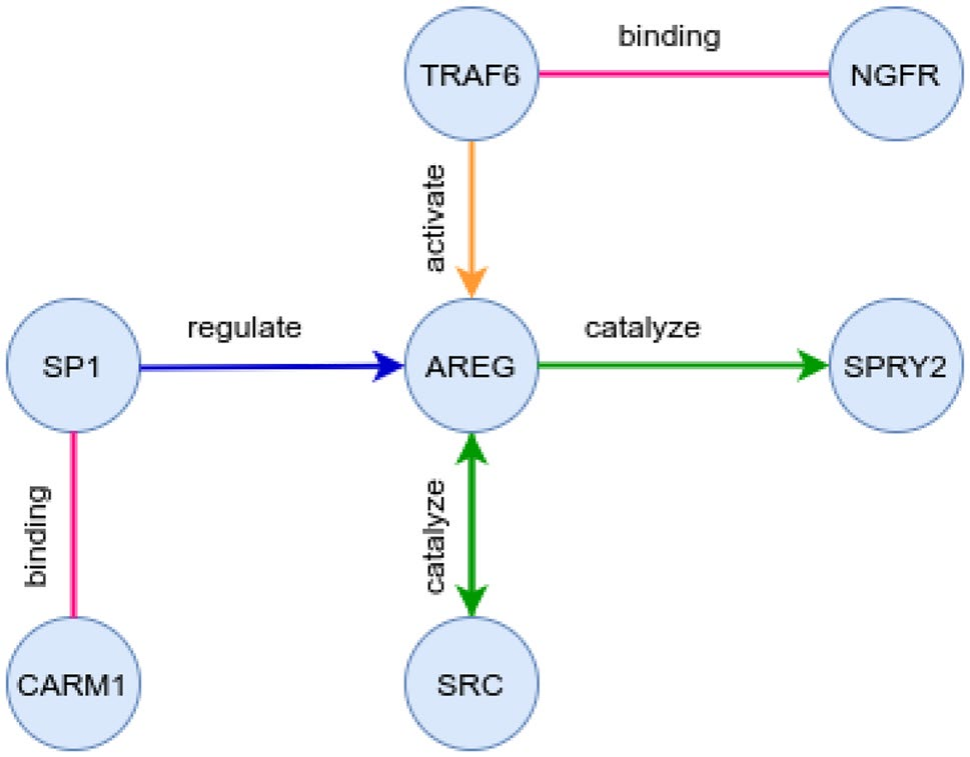
An example of the protein functional interaction network with the various relation types highlighted by different colors.

A pictorial example of the protein functional interaction network is presented in Fig. 4. Different relations are depicted using different colors. Since regulation, inhibition, catalyze, and activation are one-way relations, we model the protein functional interaction network as a directed graph. We retrieve the protein functional interaction network from^92^ (version 2020). We generate a directed graph from the given data as follows. Each PCG is represented as a node; a protein-protein binding interaction is modeled as two directed edges. Each relation, i.e., inhibits, activates, regulates, and catalyzes, is represented by a directed edge between the corresponding nodes. Overall, our protein functional interaction network consists of 423,672 directed links between 23,611 PCGs. Some PCG nodes might be isolated in the generated network because we only include experimentally verified interactions.

##### Modelling miRNAs using the protein functional interaction networks

We obtain the experimentally validated miRNA-PCG interactions from the miRTarBase database^60^ (release 8.0). We then model each miRNA as a network of PCGs built up from the protein functional interaction network. There is a directed link between two nodes if there is a directed link between the corresponding nodes in the functional interaction network. Each PCG node in the network has a feature vector of one dimension. The feature value of a PCG node is set to 1 if there is a known interaction between it and the current miRNA, and 0 otherwise.

##### Modelling diseases using the protein functional interaction networks

We obtain the disease-PCG associations from the DisGeNET^61^ database, which contains one of the largest publicly available collections of genes associated with human diseases. As above, we then model each disease as a network that contains all PCGs from the protein functional interaction network. There is a directed link between two nodes if there is a directed link between the corresponding nodes in the functional interaction network. Each PCG in the network has a feature vector of one dimension. The feature value of a PCG node is set to be the *normalized confidence score* of the corresponding association between the PCG and the current disease if there exists one, and 0 otherwise.

#### The message passing framework for feature enrichment

The message passing module is responsible for further enriching the input representations via a simple message passing technique. It takes as input the miRNAs and diseases modeled using the protein functional interaction networks with the corresponding node features as described in the previous section.

**Figure 5:**
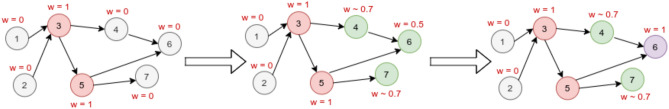
An example of how a message passing framework functions. The numbers inside the circles indicate nodes’ IDs. ‘w’ indicates the node feature weight (as described in section “The message passing framework/module”). In the first iteration, new weights for nodes 4, 6, 7 are calculated according to equation (1). Only the weight for node 6 gets updated during the second iteration.

miRNA-target or disease-PCG association data might be incomplete due to the lack of biological experiments or other technical limitations. Moreover, the data acquisition methods might fail to detect *indirect* PCG associations. Our message passing strategy allows us to infer such indirect or missing miRNA-PCG and disease-PCG connections. In particular, at each iteration, a message passing step is performed in which only weights of the nodes with unknown associations (i.e., nodes with initial 0 weights) with miRNAs/diseases are updated. Formally, the inferred weight for a particular node *i* whose original weight is 0 at iteration *t* is calculated in accordance with its parents and their degrees as follows:

1 $$\begin{aligned} \mathbf {w}_t(i) ={1\over \sqrt{ \mathbf {d}_{in}(i)}} \sum _{j \in Par(i)}\frac{ \mathbf {w}_{t-1}(j)}{\sqrt{\mathbf {d}_{out}(j)}} \end{aligned}$$

where *Par*(*i*) denotes the set of parent nodes of node *i*, $\mathbf {w}_{t-1}(j)$ is the weight of node *j* calculated at iteration $t-1$, $\mathbf {d}_{in}(i)$ and $\mathbf {d}_{out}(j)$ denote the in-degree and the out-degree of nodes *i* and *j*, respectively. We provide an example of how the proposed message passing layer/framework works in Fig. 5. The results presented in section “Results” correspond to the output from the message passing framework after one iteration. We choose one iteration as it acquires the best performance on all inductive test datasets.

### The feature selection module

#### The disease category

The MESH ontology^54^ is a well-organized vocabulary produced by the National Library of Medicine, where diseases are classified into different categories. MESH ontology can be visualized as a tree where each layer in the tree represents one level of granularity. The uppermost level represents the most general category. We obtain the disease category information from the MESH database. We assign a label to each disease that corresponds to its second-level category for “*Infection*” related diseases and its first-level category for the rest. We group all categories which have less than ten members into one common “Others” category to make the label space less sparse. In the end, each disease is assigned one of the 28 categories.

#### Feature selection with a side-supervised task

To remove redundant and noisy miRNA/disease-PCG associations, we employ another source of information (the disease categories as described in section “The feature selection module”) as input to our feature selection module. The rationale driving the feature selection step is that PCGs that are important for differentiating between diseases of different classes should also be indicative of the disease conditions and should, therefore, be important factors for the miRNA-disease association prediction problem.

Formally, we are given the set of diseases $\mathbf {D}$, their associated categories $\mathbf {C}$, and their inferred (up to *t* hop(s)) PCG association profiles $\mathbf {DP}_{t}$. We are interested in finding the top *K* most important PCG features predictive of the disease category.

As suggested in^93,94^, ReliefF^95,96^ is a competitive feature selection method for biological datasets. For that reason, we employ ReliefF to select the *K* most important PCGs. ReliefF estimates each feature’s importance according to the relationship of *n* random samples to their nearest neighbors. For a given sample, the algorithm selects *k* nearest samples from the same class (hits) and *k* nearest samples from each of the other classes (misses). The feature importance is then quantified as to how well it can differentiate between the misses and the hits samples. The results presented in section “Results” correspond to $K=100$ as it acquires the best performance on all inductive testing datasets.

### The structural embedding learning

#### **Network construction**

Let $\mathbf {P}_K$ denote the set of *K* most informative PCGs for the disease category prediction task obtained as output from the feature selection module. Let $\mathbf {A}_p$ denote the adjacency matrix generated from the subset of PCG-PCG interactions for all PCGs in $\mathbf {P}_K$. Similarly, let $\mathbf {A}_{mp}$ be the adjacency matrix generated from the subset of miRNA-PCG associations for all PCGs in $\mathbf {P}_K$. $\mathbf {A}_{dp}$ denotes the adjacency matrix generated from the subset of disease-PCG associations for all PCGs in $\mathbf {P}_K$. Let $\mathbf {A}_{md}$ be the adjacency matrix constructed from the known miRNA-disease associations. We construct an undirected network $\mathcal {G}_{mdp}$ from the training miRNA-disease associations and the filtered sets of miRNA-PCG, disease-PCG associations, and PCG-PCG interactions. The adjacency matrix for $\mathcal {G}_{mdp}$ is then given as follows:

$$\begin{aligned} \mathbf {A}_{mdp} = \left[ \begin{array}{ *{3}{c} } \mathbf {Z}_m &{} \mathbf {A}_{md}&{} \mathbf {A}_{mp} \\ \mathbf {A}_{md}^T &{} \mathbf {Z}_d &{} \mathbf {A}_{dp}\\ \mathbf {A}_{mp}^T &{} \mathbf {A}_{dp}^T &{} \mathbf {A}_p\\ \end{array} \right] \end{aligned}$$

where $\mathbf {Z}_m \in \mathbf {R}^{n_m\times n_m}$ and $\mathbf {Z}_d \in \mathbf {R}^{n_d\times n_d}$ are the matrices of all zeros; $n_m$ and $n_d$ are the number of miRNAs and diseases, respectively.

#### Structural deep network embedding

The Structural Deep Network embedding^97^ is a node representation learning method that can capture the network’s global and local structure efficiently by employing a deep autoencoder. The model is claimed to be able to learn highly non-linear network structures while being robust to the network sparsity^97^. In particular, SDNE enforces the first-order similarity constraint, which basically implies that two vertices in a network are similar if they are linked by an observed edge as a supervised signal to learn the local network structure. The second-order proximity, which assumes that two vertices sharing many common neighbors are similar, is also incorporated into the model to capture the global network structure. A comparative study presented in^19^ indicates that SDNE acquires the best performance compared with other structural embedding methods for the miRNA-disease association prediction problem. For that reason, we adapt SDNE to learn the structural embeddings for miRNAs and diseases from the $\mathcal {G}_{mdp}$ network. We use the SDNE implementation shared by^19^ to generate the embeddings for miRNAs and diseases from the inter-connected miRNA-PCG-disease network. The results presented in section “Results” correspond to the SDNE with two encoder layers of size [1000, 128], one decoder layer, and the output embedding of 128 dimensions as suggested in^19^.

### The classification module

#### The features

##### The miRNA family features

miRNAs belonging to the same family usually share a common ancestor in the phylogenetic tree. They are also believed to share similar secondary structures and have similar biological functions^98^. Consequently, miRNA family information is highly relevant to the miRNA-disease association prediction task. We retrieve the miRNA family information from mirBase database^99^. In the end, each miRNA is assigned to one of the 1375 families. We model each miRNA’s family features as the one-hot encoding of its family.

##### The disease semantic similarity features

he disease semantic similarity^20,49^ quantifies how similar two particular diseases are based on their relative positions on the disease MESH ontology^54^. We use the code and the setup in^44^ to compute a disease semantic similarity matrix for our 3679 diseases set. Each entry (i,j) in the matrix indicates how similar disease *i* is to disease *j*. We model each disease’s semantic similarity features as the corresponding row entry in the similarity matrix.

#### The classifier

**Figure 6:**
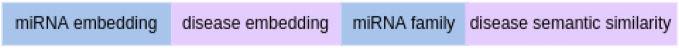
The final miRNA-disease input pair representation.

The final classifier module takes the input representation for miRNA-disease pairs and for each pair, it outputs an association probability in the [0,1] range. The higher the probability, the more likely the input pair is associated. For a particular (*m*, *d*) input pair, we construct the input feature vector as the concatenation of their corresponding structural embeddings, the miRNA family, and disease semantic similarity features. More specifically, $\mathbf {X}_{md} = [\mathbf {E}_m,\mathbf {E}_d, \mathbf {F}_m, \mathbf {S}_d]$, where ${X}_{md}$ denotes the input feature vector corresponding to (*m*, *d*); $\mathbf {E}_m,\mathbf {E}_d$ represent the pre-trained embeddings output from SDNE; while $\mathbf {F}_m$ refers to the miRNA family feature for miRNA *m*; $\mathbf {S}_d$ corresponds to the disease semantic similarity for disease *d*. A pictorial illustration of the final miRNA-disease pair representation is given in Fig. 6. We train a Random Forest classifier^100,101^ with 350 estimators to do the association prediction task.

## Supplementary Information

Supplementary Information.

## Data Availability

All the code and data are publicly available at https://git.l3s.uni-hannover.de/dong/mpm. All generated predictions and related domain information can be found at http://software.mpm.leibniz-ai-lab.de/.
