# Supplementary material for: A message passing framework with multiple data integration for miRNA-disease association prediction
Source: Sci Rep. 2022 Sep 28;12:16259. doi: 10.1038/s41598-022-20529-5 (PMC9519928; doi:10.1038/s41598-022-20529-5)
Supplement: Supplementary file 1 — Supplementary Information. [file 41598_2022_20529_MOESM1_ESM.pdf]

Supplemental materials for: "A message passing framework with multiple data integration for miRNA-Disease association prediction"

Thi Ngan Dong[1,*], Johanna Schrader[1], Stephanie Mücke[2], and Megha Khosla[3]

[*]dong@l3s.de
[1]L3S Research Center, Leibniz University of Hannover, Hannover, Germany
[2]TRAIN Omics, Translational Alliance in Lower Saxony, Hannover, Germany
[3]Delft University of Technology (TU Delft), Netherlands

ABSTRACT

In this supplementary file, we present the details regarding our compared models in Section 1. Section 2 provides the details corresponding to our data acquisition, pre-processing, benchmarked datasets generation, and negative sampling strategies. Section 3 shows the detailed experimental and ablation study's results on the 18 test sets for new diseases. Finally, in Section 4, we offer a user guide for the website where we encapsulate all our model-generated predictions for 1,618 miRNAs and 3,679 diseases and some related domain knowledge.

1 The compared models

EPMDA[1] EPMDA first builds a heterogeneous graph in which nodes are miRNAs and diseases, and the edges are built up from miRNA-miRNA, disease-disease Gaussian Interaction Profile Kernel (GIP)[2] similarities and miRNA-disease known associations). They then proposed a graph topology-based edge feature extraction technique operating on the constructed graph. The extracted features will then be fed as input to a Multiple Layer Perceptron (MLP) classifier, which will assign 1 or 0 labels to an input edge. Though EPMDA proposed a graph-based feature extraction approach, their input graph is still constructed from the miRNA and disease GIP similarities. Therefore, EPMDA is still classified as a hand-crafted-based method.

NIMGCN[3] NIMGCN proposes an end-to-end learning framework that operates on a heterogeneous network $\mathscr{G}$, which is built up from miRNA-disease known associations, the pre-calculated miRNA functional similarity (MISIM), and the disease semantic similarities. Two GCNs followed by two non-linear transformation layers are employed to learn the latent representation for miRNAs and diseases separately. Though NIMGCN's contribution lies in the graph-based feature transformation technique, the building blocks for the $\mathscr{G}$ are still pre-calculated similarities or hand-crafted features. Therefore, NIMGCN is still a hand-crafted-based approach.

DBMDA[4]. DBMDA is also a hand-crafted-based method. The model can be separated into two modules: the unsupervised feature transformation and the Rotation Forest classifier. The unsupervised feature transformation consists of two auto-encoders whose tasks are to learn the hidden representation for a miRNA-disease pair from the miRNA functional similarities (retrieved from MISIM), disease semantic similarities, and miRNA sequence similarities. The encoded representation will then be fed as input into a Rotation Forest classifier whose job is to predict potential miRNA-disease associations.

NEMII[5] NEMII employs Structural Deep Network Embedding (SDNE) to learn the embeddings for miRNAs and diseases from a bipartite graph built up from known miRNA-disease association information. The learned embedding will then be concatenated with the features extracted from the miRNA family and disease semantic similarity to form the input to a Random Forest classifier. Since NEMII still makes use of disease semantic similarities, it is a hybrid technique.

NEMII does not work effectively for large datasets with many new diseases for two main reasons. On the one hand, the Rotation Forest classifier is a tree-based ensemble model whose complexity is at least $O(mn^2 logn)$[6], where m is the feature size, and n is the number of training samples. In NEMII, feature space grows along with the number of miRNAs and diseases. For a large dataset with 3,679 diseases, NEMII is extremely expensive to run with the input feature size of several thousand. On the other hand, as new diseases appear as unconnected nodes in the bipartite input graph, NEMII cannot learn any meaningful structural embeddings for them. We do not have the results available for NEMII in the inductive test setting for those reasons.

DIMIG 2.0[7] DIMIG 2.0 is a semi-supervised approach that treats miRNA-disease association prediction as a multi-class classification problem where diseases are the labels. They do not use miRNA-disease association during the training process. Instead, they use only the known disease-PCG interactions to learn the model parameters. PCG nodes are connected with miRNA nodes in a heterogeneous network based on miRNA-PCG interactions. Learned signals are then propagated through the heterogeneous network to infer miRNAs' labels. DIMIG 2.0 is a feature learning-based approach.

MUCOMID[8] MUCOMID proposes different ways of integrating additional information sources. Similar to MPM, MUCOMID does not rely on secondary or hand-crafted features but employs graph neural networks to learn miRNA, disease, and PCG representations automatically from three information sources: the PCG-PCG interaction, miRNA family, and disease ontology. However, unlike MPM, MUCOMID does not use miRNA-PCG and disease-PCG associations to construct input features. Instead, MUCOMID incorporates such information as additional side tasks to further regularize the model. A dynamic loss balancing technique is employed to train the multitask model in an end-to-end manner. MUCOMID is a feature learning-based approach.

2 The Experimental data and setup

Table 1. The association data statistics where $|n_{md}|$, $|n_m|$, $|n_d|$ refer to the number of associations, miRNAs and diseases respectively.

DATASET	$\lvert n_{md}\rvert$	$\lvert n_m\rvert$	$\lvert n_d\rvert$
HMDD2	4,592	442	309
HMDD3	10,494	742	545
HMDD2 ∪ HMDD3	10,980	742	591
HELD-OUT	4,311	382	226
HELD-OUT2	6,388	697	509
NOVEL-MIRNA	4,734	638	227

2.1 The miRNA-disease association data source

We retrieve the set of miRNA-disease associations from the HMDD v2.0[9] and HMDD v3.0[10] databases. We then perform various pre-processing and filtering steps as described in section 2.2. In the end, the filtered data for the HMDD v2.0 database (denoted as HMDD2) contains 4,592 known associations between 442 miRNAs and 309 diseases. The filtered data for the HMDD v3.0 database (referred to as HMDD3) includes 10,494 known associations between 742 miRNAs and 545 diseases.

2.2 Data acquisition and pre-processing

As the quantity and quality of the employed data source greatly impact the predictive power of the learned models, apart from the model development, our contribution also lies in the data acquisition and pre-processing. In the following sections, we describe our data acquisition and pre-processing steps.

2.2.1 Disease ID matching

The data deposited in HMDD 2.0 and HMDD 3.0 only provides disease names. Even worse, different names might refer to the same diseases. In addition, to retrieve the disease ontology or disease-PCG associations, we need the diseases' MESH IDs. Therefore, in the first steps of our pre-processing pipeline, we match the HMDD 2.0's and HMDD 3.0's disease names with their corresponding MESH IDs.

In order to do that, we first collect the list of disease IDs, along with their names and synonyms, from the MESH database[11]. We then standardize all disease names and synonyms (remove redundant spaces and quotations, and convert all to lowercase). After that, our disease matcher works as follows: (i) if there is an exact match between the searching disease name and any MESH names/synonyms, then it assigns the corresponding MESH ID to that disease name (ii) otherwise, it outputs a list of names along with their MESH IDs which are the most similar to the searching name and only contain up to several different characters in the character sequence. We later quickly reviewed these lists to increase the data coverage as much as possible.

2.2.2 miRNA name standardization

The HMDD 2.0 and HMDD 3.0 databases store the known associations reported in scientific publications and do not reflect the changes in the miRNA knowledgebase over time. Therefore, the same miRNA might appear with different IDs in the miRNA-disease association databases. To remove unnecessary noise and make the data consistent, we standardize the miRNA IDs according to miRBase[12] - one of the most reliable and popular databases to retrieve miRNAs related information. More

specifically, we match multiple miRNAs aliases together and obsoleted IDs to the newly assigned ones according to the data retrieved from miRBase, version 22.1. Table 2 presents the statistics associated with the number of miRNAs and miRNA-disease associations after standardization.

Table 2. Statistics for miRNA-disease data before and after miRNA name standardization. n_m refers to the number of miRNAs while n_{md} denotes the number of miRNA-disease associations.

Database	Before		After	
	n_m	n_{md}	n_m	n_{md}
HMDD 2.0	578	6,401	540	5,909
HMDD 3.0	1,120	15,165	859	12,552

2.3 The transductive testing setup

The transductive testing setup aims at evaluating different models' performances on the set of partially observed miRNAs and diseases. We train each model with the HMDD2 dataset while testing them with the HELD-OUT test set as described below.

Let **M** and **D** denote the set of miRNAs and diseases observed in the HMDD2 dataset, correspondingly. We construct the HELD-OUT dataset by restricting the set of miRNAs and diseases to **M** and **D** and including only the miRNA-diseases associations, which appear in the HMDD3 dataset but not in the HMDD2 dataset. A mathematical description of HELD-OUT is given below:

$$\text{HELD-OUT} = (\mathbf{M} \times \mathbf{D} \cap \text{HMDD3}) \setminus \text{HMDD2}$$

Where $\mathbf{M} \times \mathbf{D}$ denotes the set of all possible pair combinations between miRNAs in **M** and diseases in **D**. Table 1 presents the transductive training and testing data statistics. We generate the negative training and testing samples using the negative sampling strategy given in section 2.5.

2.4 The inductive setting setup

2.4.1 The large independent testing sets

The HELD-OUT2 test set. HELD-OUT2 contains all associations that appear in HMDD3 but not in HMDD2. We devise this dataset to test all models' performance on a large independent test set that contains both new miRNAs and new diseases (with respect to the training data). After pre-processing, HELD-OUT2 contains 6,388 known associations for 697 miRNAs and 509 diseases. Among those, there are 300 new miRNAs and 282 new diseases that do not appear in the training set HMDD2.

The NOVEL-MIRNA test set. The NOVEL-MIRNA test set is a subset of the HELD-OUT2 test set. To construct NOVEL-MIRNA, we remove all associations related to any disease that does not appear in **D**. In the end, NOVEL-MIRNA contains 4,734 known associations for 638 miRNAs and 227 diseases in which there are 256 new miRNAs that do not appear in the training set HMDD2. The data statistics for our large independent test sets are presented in Table 1.

2.4.2 The datasets for new diseases

Table 3. The individual diseases' training and testing dataset statistics where $|E_{train}|$, $|E_{val}|$, $|E_{test}|$ refer to the number of *positive* training, validating and testing samples respectively.

DISEASE	$\|E_{train}\|$	$\|E_{test}\|$	DISEASE	$\|E_{train}\|$	$\|E_{test}\|$
D001943	10649	331	D005909	10803	177
D015179	10704	276	D001749	10813	167
D013274	10720	260	D012516	10821	159
D008175	10757	223	D010190	10822	158
D011471	10761	219	D006333	10838	142
D002289	10766	214	D002292	10839	141
D010051	10789	191	D003110	10854	126
D008545	10791	189	D015470	10868	112
D005910	10791	189	D002294	10876	104

The inductive setting setup aims at evaluating models' performances on completely new diseases and is described as follows:

- Let H = HMDD2 ∪ HMDD3

- We take out the set of diseases $\hat{\mathscr{D}}$ such that each disease $d \in \hat{\mathscr{D}}$ has more than 100 known associations in H. There are 18 such diseases.
- For each disease $d \in \hat{\mathscr{D}}$, a dataset is created as follows: (i) The positive training set includes all known associations in H except those associated with d, (ii) The negative training samples are generated according to section 2.5, (iii) We evaluate all models on the ***complete*** testing set where all known associations for d in H form the positive test set and the negative testing samples consist of all possible combinations of d and any miRNA that does not appear in the positive testing set.

Table 3 presents the statistics corresponding to our 18 datasets for new diseases.

2.5 The negative sampling strategy.

We define the negative pool as the set of all possible combinations of miRNA-disease pairs that do not appear in the set of all known associations. For all training data, we fix the negative:positive ratio to 1:1. For the independent testing sets (Held-out, Novel-miRNA, and Held-out2), we vary the ratio to be one of [1:1, 1:5, 1:10]. For each negative:positive sample rate, we randomly draw 10 subsets from the negative pool and evaluate all models' performance on all those sampled sets to avoid bias and make the comparison as fair as possible. In summary, in the transductive setting, we have 10 train and 10 test sets (corresponding to different negative sample sets). We evaluate each model by training it on all 100 train and test set combinations, each with 2 random model initialization. In total, we report the average results corresponding to 200 experimental runs for the transductive setting.

For the inductive setup, we use the entire set of unknown interactions as negative test samples. We run the model with 10 train sets each time with 2 random initialization of the model. For the inductive setting, we, therefore, report the average results over 20 experimental runs.

2.6 Hyperparameter setup and implementation details

MPM and its variants. We experiment with the number of message passing iteration t in [1, 2, 10]. For the feature selection module, we run ReliefF[13] with 20 neighbors and the number of selected features K from 50 to 500 with a step size of 50. The results reported in Section 2 in the main paper correspond to $t = 1$, and $K = 100$, which result in the best average AP score among 18 datasets in the inductive test setting. For SDNE, we use the default parameter as suggested by NEMII[5] with the embedding size fixed to 128. The Random Forest classifier is trained with 350 estimators.

Existing benchmarked models. For EPMDA, DBMDA, and NIMGCN, we use the code and setup released in[14]. For NEMII and MuCoMiD, we use the same code and setup as published by the authors. For DimiG 2.0, we follow the same testing strategies employed in[8].

3 Details experiment results

3.1 MPM with different binary classifiers

Table 4. MPM with different binary classifiers results on the 18 inductive testing dataset for new diseases.

Dataset	Classifier	AUC	AP	SN	SP	Acc	Pre	F1	Mcc	Top100
D001943	SVM	**0.9064**	**0.8288**	82.16	87.88	86.71	64.15	**71.82**	64.36	97.8
	Random Forest	0.8954	0.8239	55.65	**98.63**	**89.84**	**91.35**	69.05	**66.26**	**99.4**
	AdaBoost	0.8919	0.7875	78.25	74.71	75.43	65.0	65.48	53.95	96.55
	K-Nearest Neighbors	0.8376	0.6154	68.19	88.84	84.62	61.42	64.53	54.95	84.6
	Gaussian Naive Bayes	0.5108	0.2071	76.59	24.83	35.42	20.76	32.67	1.33	20.7
	MLP	0.8055	0.6984	69.2	68.01	68.25	52.45	51.96	38.95	92.5
	Decision Tree	0.8385	0.6539	**91.6**	28.07	41.06	30.59	42.63	17.47	92.9
D015179	SVM	**0.9128**	0.7975	**90.67**	66.62	70.72	37.13	52.3	44.35	92.1
	Random Forest	0.9052	**0.806**	74.22	95.07	**91.51**	75.64	**74.9**	**69.81**	**95.15**
	AdaBoost	0.8923	0.7697	51.96	95.84	88.35	82.72	55.94	55.64	93.9
	K-Nearest Neighbors	0.8412	0.5136	83.88	71.77	73.83	41.03	54.16	45.08	65.55
	Gaussian Naive Bayes	0.5337	0.1811	86.92	19.64	31.12	18.2	30.09	6.36	18.55
	MLP	0.8361	0.6852	87.16	45.63	52.71	28.94	41.84	26.73	86.7
	Decision Tree	0.8643	0.6896	55.25	**98.0**	90.7	**86.27**	65.84	63.67	89.55

D013274	SVM	**0.9491**	**0.8413**	70.23	97.03	**92.72**	81.92	**75.6**	**71.66**	95.4
	Random Forest	0.9379	0.8365	82.0	92.7	90.98	68.29	74.5	69.52	**96.0**
	AdaBoost	0.9257	0.7897	79.35	89.67	88.01	67.46	70.12	65.56	94.3
	K-Nearest Neighbors	0.8076	0.4311	85.69	58.65	63.0	29.7	43.7	33.37	56.15
	Gaussian Naive Bayes	0.5641	0.1805	**90.38**	22.25	33.2	18.2	30.31	11.55	18.9
	MLP	0.9051	0.7856	40.54	**99.18**	89.76	**91.08**	55.52	56.36	92.3
	Decision Tree	0.8245	0.5623	83.35	69.19	71.46	44.63	55.21	44.66	64.9
D008175	SVM	**0.9358**	**0.7677**	95.38	63.42	67.82	30.8	46.2	41.77	87.5
	Random Forest	0.9263	0.764	92.31	76.5	78.68	39.42	55.01	51.01	**87.6**
	AdaBoost	0.9211	0.7308	**97.53**	20.69	31.28	20.65	32.16	13.73	85.6
	K-Nearest Neighbors	0.8483	0.5849	71.21	**94.69**	**91.45**	**68.26**	**69.67**	**64.74**	78.15
	Gaussian Naive Bayes	0.5626	0.1548	91.88	20.51	30.35	15.6	26.67	10.94	17.1
	MLP	0.8594	0.6532	89.62	42.76	49.22	26.6	38.12	25.36	80.1
	Decision Tree	0.8153	0.5184	92.74	42.61	49.52	26.63	38.62	25.01	67.7
D011471	SVM	**0.9334**	**0.776**	59.13	**97.19**	**92.04**	**76.85**	66.76	63.06	**91.1**
	Random Forest	0.9222	0.7329	80.14	91.48	89.95	59.59	**68.34**	**63.5**	85.45
	AdaBoost	0.9084	0.7174	56.9	96.58	91.2	66.48	60.03	56.82	84.65
	K-Nearest Neighbors	0.8821	0.5619	75.85	90.99	88.94	56.89	65.0	59.45	73.15
	Gaussian Naive Bayes	0.5548	0.1498	**87.58**	23.0	31.74	15.11	25.78	8.8	14.45
	MLP	0.8815	0.6685	49.13	96.75	90.3	72.88	56.43	53.79	79.6
	Decision Tree	0.8642	0.6052	78.45	83.51	82.82	58.44	64.22	57.51	77.5
D002289	SVM	**0.9428**	0.8002	55.4	98.35	**92.67**	83.71	66.66	64.43	90.9
	Random Forest	0.9341	**0.8024**	40.0	**99.49**	91.62	**92.35**	55.65	57.48	**92.1**
	AdaBoost	0.9378	0.7562	69.53	96.15	92.63	74.12	**71.09**	**67.33**	86.8
	K-Nearest Neighbors	0.7691	0.4	42.15	94.77	87.81	55.84	47.9	41.75	61.5
	Gaussian Naive Bayes	0.567	0.15	**92.66**	20.49	30.04	15.08	25.95	11.42	14.9
	MLP	0.8688	0.6634	62.62	90.15	86.51	62.59	58.47	53.82	80.0
	Decision Tree	0.8576	0.6043	41.12	98.02	90.5	81.29	51.0	51.55	72.6
D010051	SVM	**0.9644**	**0.7977**	90.34	91.28	91.17	58.23	70.76	68.09	**88.9**
	Random Forest	0.9575	0.7921	87.85	92.48	**91.93**	61.05	**72.01**	**69.02**	87.1
	AdaBoost	0.9525	0.7508	84.03	**92.99**	**91.93**	**63.24**	71.57	68.38	84.55
	K-Nearest Neighbors	0.8859	0.5064	88.38	76.66	78.04	34.19	49.12	45.72	60.7
	Gaussian Naive Bayes	0.5882	0.14	**95.34**	22.03	30.69	14.07	24.51	14.02	14.65
	MLP	0.9319	0.7493	84.58	86.81	86.55	52.5	62.68	59.42	85.15
	Decision Tree	0.9073	0.6246	74.35	84.35	83.17	60.51	59.05	55.27	72.2
D008545	SVM	**0.9341**	**0.7524**	50.48	98.17	92.59	78.68	61.39	59.31	**83.7**
	Random Forest	0.9168	0.7239	52.09	97.98	**92.62**	77.57	62.12	**59.75**	82.45
	AdaBoost	0.9218	0.6877	39.1	97.87	91.0	**84.85**	47.49	50.59	80.85
	K-Nearest Neighbors	0.8453	0.5134	65.66	94.23	90.9	60.1	**62.75**	57.65	70.75
	Gaussian Naive Bayes	0.5635	0.1319	**90.59**	22.11	30.11	13.33	23.24	10.08	11.25
	MLP	0.8858	0.6408	21.88	**99.22**	90.18	80.78	32.11	36.85	75.5
	Decision Tree	0.8827	0.577	61.96	95.78	91.83	70.42	62.14	59.94	71.9
D005910	SVM	**0.9597**	**0.7809**	79.58	96.4	**94.43**	74.54	**76.95**	**73.86**	**83.8**
	Random Forest	0.9473	0.7592	48.97	**98.41**	92.63	80.93	59.28	58.55	81.6
	AdaBoost	0.9452	0.7413	49.42	98.2	92.5	**81.19**	58.24	58.22	81.95
	K-Nearest Neighbors	0.8923	0.6218	74.5	95.66	93.19	69.82	71.94	68.22	78.0
	Gaussian Naive Bayes	0.5894	0.139	**95.88**	21.86	30.51	13.96	24.37	14.3	13.5
	MLP	0.8916	0.6461	60.74	94.45	90.52	62.39	59.72	55.55	75.1
	Decision Tree	0.9168	0.6065	63.17	93.7	90.14	46.09	52.64	49.72	61.45
D005909	SVM	**0.945**	0.7336	70.9	96.03	93.29	68.74	69.78	66.03	79.2
	Random Forest	0.9378	**0.7361**	75.14	95.57	**93.34**	67.64	**71.14**	**67.54**	**79.7**
	AdaBoost	0.9348	0.678	73.28	94.59	92.26	64.03	66.71	63.56	77.0
	K-Nearest Neighbors	0.8786	0.5423	69.44	94.44	91.7	60.78	64.73	60.29	70.15
	Gaussian Naive Bayes	0.5826	0.1286	**94.86**	21.8	29.8	12.97	22.82	13.01	13.65

	MLP	0.8728	0.5372	39.75	**96.55**	90.34	61.2	45.7	43.25	62.7
	Decision Tree	0.9019	0.5579	77.12	91.26	89.71	47.54	58.48	55.87	63.95
D001749	SVM	**0.9471**	0.7589	85.93	87.44	87.29	44.2	58.32	55.75	85.0
	Random Forest	0.9403	**0.7845**	88.32	84.7	85.08	40.09	55.09	53.03	**88.65**
	AdaBoost	0.9285	0.7118	89.94	60.25	63.31	33.63	45.49	38.12	84.0
	K-Nearest Neighbors	0.9087	0.5276	84.43	**91.13**	**90.44**	**52.29**	**64.58**	**61.69**	60.8
	Gaussian Naive Bayes	0.5597	0.1157	88.74	22.59	29.42	11.66	20.61	8.41	11.45
	MLP	0.9049	0.6473	73.8	90.73	88.98	52.22	59.74	55.86	73.9
	Decision Tree	0.7784	0.4111	**90.78**	55.64	59.27	30.75	41.33	35.8	45.35
D012516	SVM	**0.9429**	**0.7141**	43.11	98.94	93.46	81.73	56.4	56.45	77.6
	Random Forest	0.9319	0.7131	43.96	98.98	**93.57**	82.6	57.28	57.37	**78.8**
	AdaBoost	0.929	0.6531	48.87	97.98	93.16	78.11	57.49	57.28	76.1
	K-Nearest Neighbors	0.8435	0.5023	52.64	97.11	92.74	66.49	58.74	55.28	68.4
	Gaussian Naive Bayes	0.5782	0.1147	**90.69**	24.58	31.08	11.59	20.55	10.79	11.05
	MLP	0.8619	0.5416	49.81	95.85	91.32	58.13	52.82	48.75	63.85
	Decision Tree	0.9016	0.5967	52.39	97.76	93.3	74.36	**60.29**	**58.49**	71.95
D010190	SVM	**0.9487**	**0.7912**	66.9	97.42	**94.44**	73.92	70.17	67.26	**84.2**
	Random Forest	0.9437	0.7494	83.1	94.2	93.12	60.88	**70.23**	**67.5**	79.7
	AdaBoost	0.9388	0.7327	47.66	98.3	93.36	**84.99**	53.72	56.28	80.65
	K-Nearest Neighbors	0.9208	0.561	87.72	91.24	90.9	52.03	65.31	63.2	64.7
	Gaussian Naive Bayes	0.5786	0.1141	**94.3**	21.4	28.52	11.49	20.49	11.68	11.4
	MLP	0.9192	0.6717	39.05	98.45	92.66	76.54	49.82	50.37	72.2
	Decision Tree	0.9177	0.5976	86.46	91.5	91.01	57.49	68.3	65.91	68.85
D006333	SVM	**0.9562**	**0.6753**	70.35	95.46	93.25	60.06	**64.68**	**61.29**	**70.5**
	Random Forest	0.9491	0.669	67.22	95.9	**93.38**	61.23	64.04	60.51	68.8
	AdaBoost	0.9511	0.6211	47.04	**97.32**	92.91	**67.23**	51.86	51.1	66.6
	K-Nearest Neighbors	0.8476	0.4902	65.7	95.26	92.66	57.26	61.15	57.31	65.9
	Gaussian Naive Bayes	0.617	0.1116	**98.59**	24.37	30.88	11.14	20.02	15.59	10.95
	MLP	0.8158	0.5015	46.66	96.39	92.02	57.73	50.25	47.08	60.8
	Decision Tree	0.9092	0.5854	65.77	95.75	93.12	61.43	62.44	59.4	67.9
D002292	SVM	**0.9441**	0.6616	85.25	88.92	88.6	42.49	56.66	55.08	67.6
	Random Forest	0.939	**0.6838**	80.03	93.05	91.91	52.43	63.32	60.69	**70.8**
	AdaBoost	0.9212	0.6207	88.51	75.54	76.68	36.88	49.6	46.89	67.1
	K-Nearest Neighbors	0.8914	0.4157	86.38	80.07	80.62	29.75	44.1	43.2	51.6
	Gaussian Naive Bayes	0.5769	0.1016	**93.62**	21.48	27.76	10.22	18.43	10.61	10.35
	MLP	0.9126	0.5725	79.08	85.95	85.35	41.56	52.44	50.11	62.15
	Decision Tree	0.8974	0.4884	81.42	**93.11**	**92.09**	**53.09**	**64.24**	**61.77**	58.05
D003110	SVM	0.9427	0.5493	**99.6**	26.7	32.37	10.95	19.6	16.5	59.4
	Random Forest	**0.9453**	**0.659**	99.21	19.64	25.83	9.74	17.69	12.64	**68.3**
	AdaBoost	0.9393	0.6361	96.19	47.49	51.28	20.19	31.79	26.94	66.45
	K-Nearest Neighbors	0.5971	0.0972	98.33	6.69	13.83	8.18	15.1	5.28	11.1
	Gaussian Naive Bayes	0.5775	0.091	95.4	19.85	25.73	9.13	16.67	10.49	8.1
	MLP	0.8878	0.4853	98.93	10.82	17.68	8.62	15.85	8.07	53.15
	Decision Tree	0.8139	0.3614	90.71	**62.72**	**64.9**	**28.59**	**40.23**	**37.71**	39.5
D015470	SVM	0.9531	0.6395	73.84	94.28	92.87	49.08	58.93	56.6	**64.2**
	Random Forest	**0.9533**	**0.6545**	70.89	**95.27**	**93.59**	**52.84**	**60.47**	**57.84**	63.4
	AdaBoost	0.9439	0.5965	83.3	91.29	90.73	42.91	55.89	55.4	60.1
	K-Nearest Neighbors	0.9149	0.4567	80.18	92.68	91.81	44.91	57.55	56.19	54.85
	Gaussian Naive Bayes	0.589	0.0831	**96.43**	21.17	26.38	8.34	15.35	11.18	7.6
	MLP	0.9114	0.5623	69.64	92.12	90.57	45.06	53.44	51.0	57.3
	Decision Tree	0.921	0.4254	81.69	92.17	91.44	44.51	56.93	56.12	48.65
D002294	SVM	**0.9624**	0.647	93.46	91.14	91.29	42.1	58.02	59.32	61.8
	Random Forest	0.9623	**0.6688**	90.63	**92.49**	**92.37**	**45.4**	**60.46**	**60.94**	**62.8**
	AdaBoost	0.9519	0.6005	86.73	91.24	90.94	43.56	55.95	56.79	60.75
	K-Nearest Neighbors	0.915	0.4008	85.48	89.74	89.47	36.42	51.08	51.55	47.55

Gaussian Naive Bayes	0.5898	0.0774	**97.12**	20.83	25.74	7.77	14.39	11.07	6.25
MLP	0.9278	0.564	83.56	89.37	89.0	39.52	52.3	52.36	56.45
Decision Tree	0.9364	0.465	91.25	90.38	90.43	39.98	55.43	56.59	52.05

This section presents an ablation study regarding MPM's performance with different binary classification models. In addition to the originally proposed model (with Random Forest), we also report the performance of MPM with the following classifiers: SVM[15], AdaBoost[16], K-Nearest Neighbors[17], Gaussian Naive Bayes[18], Multi-layer Perceptron[19] (MLP), and Decision Tree[20]. For all added binary classifiers, we use the default parameter sets. Table 4 presents the results corresponding to MPM with different binary classifiers on the 18 test sets for new diseases, averaged over 20 experimental runs. Looking at the results, we see that the Random Forest model results in the highest Precision, F1, and Top100 scores in 11 out of the 18 test sets. These results support our choice of architecture.

3.2 MPM vs. existing methods detailed results on the 18 complete test sets for new diseases.

Table 5. Benchmarked models full results on the 18 inductive testing sets for new diseases.

Dataset	Method	AUC	AP	SN	SP	Acc	Pre	F1	Mcc	Top100
	MPM	0.8954	0.8239	55.65	98.63	**89.84**	**91.35**	**69.05**	**66.26**	**99.4**
	NimGCN	0.3439	0.1595	0.0	**99.44**	79.1	0.0	0.0	-2.25	0.0
	DBMDA	0.7728	0.5067	62.42	84.77	80.2	56.0	57.92	46.28	67.1
D001943	NEMII	**0.9163**	**0.8267**	52.51	98.64	89.2	91.07	66.34	63.89	98.4
	MuCoMiD	0.6691	0.4139	42.84	75.17	68.56	42.76	36.14	21.14	52.8
	DimiG 2.0	0.5	0.2052	**100.0**	0.0	20.46	20.46	33.97	0.0	20.0
	MPM	0.9052	**0.806**	74.22	95.07	**91.51**	**75.64**	**74.9**	**69.81**	**95.15**
	NimGCN	0.3725	0.1338	0.0	**98.86**	81.99	0.0	0.0	-2.63	0.0
	DBMDA	0.7916	0.4628	77.07	73.52	74.12	44.3	54.77	43.29	53.8
D015179	NEMII	**0.9103**	0.7969	73.44	94.52	90.92	73.39	73.41	67.94	94.7
	MuCoMiD	0.6508	0.3399	76.05	49.51	54.04	25.82	37.64	20.32	41.3
	DimiG 2.0	0.4985	0.1706	**100.0**	0.0	17.06	17.06	29.14	0.0	11.5
	MPM	0.9379	**0.8365**	82.0	92.7	**90.98**	**68.29**	74.5	69.52	**96.0**
	NimGCN	0.3944	0.1323	0.0	**99.92**	83.86	0.0	0.0	-0.77	0.0
	DBMDA	0.8721	0.5031	83.96	84.23	84.18	50.9	63.28	56.81	54.5
D013274	NEMII	**0.9516**	0.8345	83.81	92.13	90.79	67.16	**74.53**	**69.65**	94.5
	MuCoMiD	0.5972	0.2492	69.77	46.67	50.38	21.01	31.58	12.94	28.8
	DimiG 2.0	0.4982	0.1607	**100.0**	0.0	16.07	16.07	27.69	0.0	18.5
	MPM	0.9263	**0.764**	92.31	76.5	78.68	39.42	55.01	51.01	**87.6**
	NimGCN	0.3736	0.1154	0.0	**97.88**	84.39	0.0	0.0	-4.11	0.0
	DBMDA	0.8289	0.4368	74.22	84.04	82.68	44.73	55.2	48.17	52.4
D008175	NEMII	**0.9352**	0.7489	88.52	84.92	**85.42**	**48.44**	**62.6**	**58.33**	85.3
	MuCoMiD	0.7344	0.3746	55.56	72.36	70.05	37.29	36.7	27.38	43.4
	DimiG 2.0	0.4986	0.1378	**100.0**	0.0	13.78	13.78	24.23	0.0	13.0
	MPM	0.9222	**0.7329**	80.14	91.48	89.95	59.59	68.34	63.5	**85.45**
	NimGCN	0.4043	0.116	0.0	**99.13**	85.71	0.0	0.0	-2.14	0.0
	DBMDA	0.7468	0.3949	64.84	74.98	73.61	40.42	47.35	35.6	52.6
D011471	NEMII	**0.9257**	0.6532	78.17	92.19	**90.29**	**61.13**	**68.55**	**63.62**	72.2
	MuCoMiD	0.6919	0.3295	42.33	71.19	67.28	38.76	21.8	15.24	39.1
	DimiG 2.0	0.4988	0.1354	**100.0**	0.0	13.54	13.54	23.84	0.0	14.5
	MPM	0.9341	**0.8024**	40.0	**99.49**	91.62	**92.35**	55.65	57.48	**92.1**
	NimGCN	0.385	0.1082	0.0	99.33	86.19	0.0	0.0	-2.14	0.0
	DBMDA	0.6609	0.3028	62.94	64.12	63.97	33.01	39.67	24.88	39.8
D002289	NEMII	**0.9466**	0.7998	59.86	97.95	**92.91**	81.86	**69.01**	**66.22**	90.1
	MuCoMiD	0.6758	0.2775	61.08	55.92	56.6	28.34	27.74	16.6	31.5
	DimiG 2.0	0.498	0.1323	**100.0**	0.0	13.23	13.23	23.36	0.0	10.5
	MPM	0.9575	**0.7921**	87.85	92.48	91.93	61.05	**72.01**	**69.02**	**87.1**
	NimGCN	0.4781	0.1139	0.0	**99.93**	88.14	0.0	0.0	-0.62	0.0

D010051

	DBMDA	0.8452	0.4002	81.41	79.01	79.3	40.07	52.89	46.82	46.3
	NEMII	**0.9599**	0.7596	54.66	97.93	**92.82**	**79.6**	63.65	61.85	84.7
	MuCoMiD	0.7738	0.3876	81.36	58.95	61.59	24.05	35.76	28.3	45.5
	DIMIG 2.0	0.4984	0.118	**100.0**	0.0	11.8	11.8	21.12	0.0	10.5
	MPM	0.9168	**0.7239**	52.09	97.98	**92.62**	77.57	**62.12**	**59.75**	**82.45**
	NIMGCN	0.4657	0.1076	0.0	**99.94**	88.27	0.0	0.0	-0.59	0.0
	DBMDA	0.7664	0.3548	70.42	69.88	69.94	33.35	42.35	32.92	44.6
D008545	NEMII	**0.9276**	0.7055	31.38	99.29	91.36	**86.14**	45.29	48.38	79.5
	MuCoMiD	0.7546	0.3652	52.49	72.21	69.91	38.74	31.38	25.59	43.4
	DIMIG 2.0	0.4986	0.1168	**100.0**	0.0	11.68	11.68	20.92	0.0	8.0
	MPM	0.9473	**0.7592**	48.97	98.41	**92.63**	**80.93**	59.28	**58.55**	**81.6**
	NIMGCN	0.4889	0.1119	0.0	**99.94**	88.27	0.0	0.0	-0.57	0.0
	DBMDA	0.6435	0.2461	36.51	83.91	78.38	30.48	31.85	20.74	32.8
D005910	NEMII	**0.953**	0.7305	50.74	98.08	92.55	77.81	**59.98**	58.47	80.1
	MuCoMiD	0.7859	0.4094	32.17	88.83	82.21	46.0	28.27	24.59	48.0
	DIMIG 2.0	0.4988	0.1168	**100.0**	0.0	11.68	11.68	20.92	0.0	10.0
	MPM	0.9378	**0.7361**	75.14	95.57	**93.34**	**67.64**	**71.14**	**67.54**	79.7
	NIMGCN	0.5634	0.1232	0.0	**99.9**	88.97	0.0	0.0	-0.76	0.0
	DBMDA	0.814	0.3694	82.04	67.9	69.44	30.66	42.86	36.38	42.5
D005909	NEMII	**0.9428**	0.7116	86.67	89.06	88.79	49.4	62.89	60.02	78.1
	MuCoMiD	0.815	0.4182	55.37	84.88	81.66	38.34	42.1	35.25	46.5
	DIMIG 2.0	0.4988	0.1094	**100.0**	0.0	10.94	10.94	19.72	0.0	12.0
	MPM	0.9403	**0.7845**	88.32	84.7	85.08	40.09	55.09	53.03	**88.65**
	NIMGCN	0.4317	0.0885	0.0	**99.85**	89.55	0.0	0.0	-0.92	0.0
	DBMDA	0.8146	0.3398	76.29	75.58	75.66	31.73	43.76	37.88	40.2
D001749	NEMII	**0.9477**	0.7668	80.84	91.39	**90.3**	**52.1**	**63.29**	**59.92**	86.4
	MuCoMiD	0.8077	0.4463	43.05	90.65	85.74	52.0	39.0	36.43	52.1
	DIMIG 2.0	0.4984	0.1032	**100.0**	0.0	10.32	10.32	18.71	0.0	12.5
	MPM	0.9319	**0.7131**	43.96	98.98	**93.57**	**82.6**	**57.28**	**57.37**	**78.8**
	NIMGCN	0.8212	0.2616	0.0	**99.93**	90.11	0.0	0.0	-0.51	6.45
	DBMDA	0.7553	0.3234	55.6	87.73	84.58	36.88	43.55	36.84	47.6
D012516	NEMII	**0.9362**	0.6583	42.32	98.33	92.82	73.49	53.24	52.16	73.4
	MuCoMiD	0.7811	0.3488	61.26	79.48	77.69	30.49	38.24	31.64	40.7
	DIMIG 2.0	0.4985	0.0983	**100.0**	0.0	9.83	9.83	17.9	0.0	13.5
	MPM	0.9437	**0.7494**	83.1	94.2	93.12	60.88	**70.23**	**67.5**	**79.7**
	NIMGCN	0.4479	0.088	0.0	**99.8**	90.05	0.0	0.0	-1.03	0.0
	DBMDA	0.871	0.3656	84.43	81.96	82.2	33.84	48.27	45.95	39.6
D010190	NEMII	**0.9466**	0.744	71.65	95.97	**93.6**	**66.23**	68.54	65.23	76.7
	MuCoMiD	0.7841	0.3726	18.55	96.82	89.18	52.07	23.65	23.54	44.7
	DIMIG 2.0	0.4986	0.0976	**100.0**	0.0	9.77	9.76	17.79	0.0	9.0
	MPM	0.9491	**0.669**	67.22	95.9	**93.38**	**61.23**	**64.04**	**60.51**	**68.8**
	NIMGCN	0.7287	0.1795	0.0	**99.94**	91.16	0.0	0.0	-0.48	4.2
	DBMDA	0.743	0.2988	58.45	85.89	83.48	32.5	41.0	35.08	44.1
D006333	NEMII	**0.9525**	0.6506	60.0	96.18	93.01	60.18	60.01	56.23	65.9
	MuCoMiD	0.8157	0.3946	36.48	88.33	83.78	45.97	29.3	28.12	45.8
	DIMIG 2.0	0.4988	0.0878	**100.0**	0.0	8.78	8.78	16.14	0.0	6.0
	MPM	0.939	**0.6838**	80.03	93.05	91.91	52.43	**63.32**	**60.69**	**70.8**
	NIMGCN	0.471	0.0818	0.0	**99.9**	91.2	0.0	0.0	-0.57	0.0
	DBMDA	0.7386	0.2383	52.48	84.4	81.62	27.8	35.55	28.72	32.5
D002292	NEMII	**0.9453**	0.6529	74.26	93.62	**91.93**	**52.75**	61.55	58.32	67.0
	MuCoMiD	0.7737	0.285	86.45	53.18	56.08	16.67	27.43	23.77	31.8
	DIMIG 2.0	0.4975	0.0871	**100.0**	0.0	8.71	8.71	16.03	0.0	5.5
	MPM	**0.9453**	**0.659**	99.21	19.64	25.83	9.74	17.69	12.64	**68.3**
	NIMGCN	0.4407	0.0687	0.0	**99.55**	**91.79**	0.0	0.0	-1.22	0.0
	DBMDA	0.8231	0.2419	91.27	70.43	72.05	23.49	36.81	37.16	23.8

D003110

	NEMII	0.9377	0.5997	90.95	82.51	83.17	**30.72**	**45.86**	**46.78**	60.8
	MuCoMiD	0.7636	0.271	97.38	13.19	19.75	8.76	16.06	7.52	31.0
	DiMiG 2.0	0.498	0.0779	**100.0**	0.0	7.79	7.79	14.45	0.0	7.5
D015470	MPM	**0.9533**	0.6545	70.89	95.27	93.59	52.84	60.47	57.84	63.4
	NimGCN	0.7322	0.158	0.0	**99.9**	92.98	0.0	0.0	-0.62	3.9
	DBMDA	0.7367	0.2588	64.02	74.17	73.46	23.8	32.79	27.35	36.1
	NEMII	0.9513	0.6251	53.39	97.44	**94.39**	**61.59**	56.69	54.15	60.6
	MuCoMiD	0.7785	0.2897	46.52	82.92	80.4	24.19	27.29	22.77	29.6
	DiMiG 2.0	0.498	0.0692	**100.0**	0.0	6.92	6.92	12.95	0.0	8.5
D002294	MPM	**0.9623**	**0.6688**	90.63	92.49	92.37	**45.4**	**60.46**	**60.94**	**62.8**
	NimGCN	0.8336	0.1855	0.0	**99.89**	**93.47**	0.0	0.0	-0.58	0.55
	DBMDA	0.7893	0.2405	68.56	80.8	80.01	22.79	33.3	31.26	31.8
	NEMII	0.9557	0.608	90.1	90.83	90.78	40.37	55.72	56.61	59.5
	MuCoMiD	0.8548	0.3842	90.86	62.46	64.29	19.72	30.18	31.1	38.7
	DiMiG 2.0	0.4982	0.0643	**100.0**	0.0	6.43	6.43	12.08	0.0	8.5

Table 5 presents the full results with all reported metrics for benchmarked models on the 18 complete test sets for new diseases, averaged over 20 experimental runs for each model. MPM gains the highest Top100 score in all test sets and the highest AP scores in 17 out of the 18 test sets.

4 An easy-to-use web application

4.1 Biological related features to support biologist justification and verification

As the associated pathway information is more intuitive compared with the list of associated PCGs, we perform pathway and functional enrichment analysis on the list of interacting/associated PCGs for each miRNA/disease and encapsulate the corresponding information into our Windows application. We perform pathway enrichment analysis by using the API provided by Reactome[21] and functional enrichment analysis by using the goscripts package[22]. We retain only pathways and GO terms whose p-values are smaller than 0.05.

4.2 The user guide

We provide an easy-to-use web application (web app) to query MPM's predictions and additional information on the miRNA, disease, and pathway data used in this work: http://software.mpm.leibniz-ai-lab.de/. In the following section, we briefly present a user guide and the functionality of the provided application.

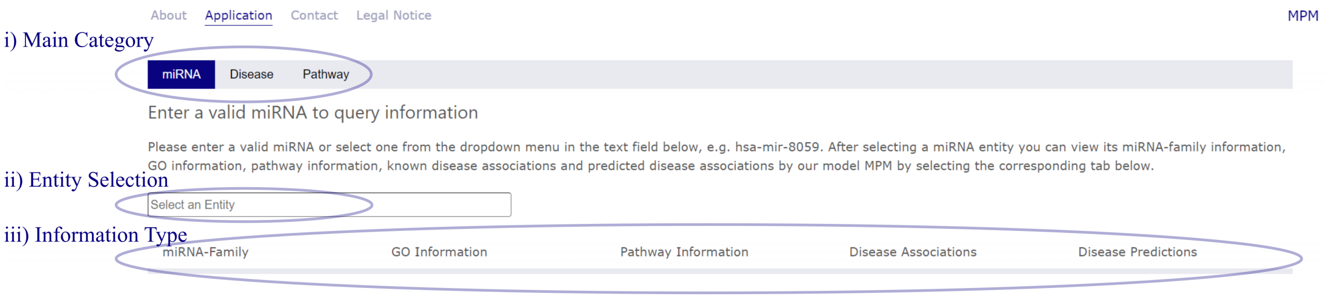

Figure 1. Web app: i) Main Category: First, the user selects whether they want to inspect a miRNA, disease, or pathway by clicking on the corresponding tab at the top of the application. ii) Entity Selection: Then, they choose a specific entity to inspect. Either the user selects one from the drop-down menu or types in the text field using auto-completion. iii) Information Type: Finally, the user selects the information they want to display by selecting the corresponding tab.

Figure 1 shows the start screen when opening the application tab in the web app and illustrates the main steps to use it. First, the user selects the **i) Main Category** from the tabs at the top of the application, i.e. *miRNA*, *Disease* or *Pathway*, marked with i) in Figure 1. In the next step **ii) Entity Selection**, the user selects a specific entity from that main category by either typing a valid entity name in the search field or by selecting an entity from the drop-down menu. The drop-down menu (which also serves as a search field) is marked with ii) in Figure 1 and opens upon selection. After a specific entity to inspect is selected, the user chooses the **iii) Information Type** they want to display by selecting the corresponding tab, marked with iii) in Figure 1.

Inspecting miRNAs

If the user wants to inspect a specific miRNA, they can choose from *miRNA-Family*, *GO information*, *Pathway Information*, *Disease Associations* (which shows confirmed associations from the data) or *Disease Predictions* (which shows associations that are predicted by MPM) to query the desired information type. The user selects the information type to query by pressing the corresponding tab from the bar below the text field, marked with ii) in Figure 1. *miRNA-Family* will display all miRNAs that belong to the same family as the selected miRNA entity. *GO information* provides the GO ID with its corresponding name and the belonging p-value for the selected miRNA entity. *Pathway information* will display all pathways and their names that the selected miRNA entity is occurring in. The pathway information additionally provides the corresponding p-value for that miRNA entity in each pathway. The GO and pathway information are sorted ascending by their p-value. The *Disease Associations* option displays all diseases associated with the selected miRNA entity that are known associations from the data. The MeSH ID with the corresponding disease name is provided. Finally, the *Disease Predictions* option provides the predictions made by MPM for the selected miRNA entity. Each predicted associated disease is displayed with its MeSH ID, disease name, and the confidence score of that prediction in descending order. Additionally, the column *Confirmed Association* shows if this specific association was known before, indicated by *yes* and - otherwise.

Inspecting Diseases

A disease can be inspected analogously to a miRNA. Similar to the miRNA category, after selecting a specific disease entity, the disease category as well allows the user to display the *GO Information*, *Pathway Information*, confirmed *miRNA Associations* to the selected disease entity as well as *miRNA Predictions* for the selected disease entity made by MPM. Contrary to the miRNA family information in the miRNA category, in the disease category, the user can display information on the *Disease Ontology*, i.e., the child and parent diseases of the selected disease entity. Example output for predictions on miRNA associations to the disease *Amyloidosis* by MPM is shown in Figure 2. The predicted associated miRNAs are shown in the left column, with their corresponding confidence score in the middle column. The right column indicates whether this association was known from the data before by *yes* or - otherwise.

Inspecting Pathways

When inspecting specific pathways, the user can choose between displaying the most significant miRNAs or diseases corresponding to the selected pathway entity. Figure 3 shows an example query for the pathway *Establishment of Sister Chromatid Cohesion*s most significant *Disease Associations*. The diseases are sorted ascending by their p-value in the right column, with the corresponding disease ID in the left and the disease name in the middle column.

References

1. Dong, Y., Sun, Y., Qin, C. & Zhu, W. Epmda: Edge perturbation based method for mirna-disease association prediction. *IEEE/ACM Transactions on Comput. Biol. Bioinforma.* (2019).
2. van Laarhoven, T., Nabuurs, S. B. & Marchiori, E. Gaussian interaction profile kernels for predicting drug–target interaction. *Bioinformatics* **27**, 3036–3043 (2011).
3. Li, J. *et al.* Neural inductive matrix completion with graph convolutional networks for mirna-disease association prediction. *Bioinformatics* (2020).
4. Zheng, K. *et al.* Dbmda: A unified embedding for sequence-based mirna similarity measure with applications to predict and validate mirna-disease associations. *Mol. Ther. Acids* **19**, 602–611 (2020).
5. Gong, Y., Niu, Y., Zhang, W. & Li, X. A network embedding-based multiple information integration method for the mirna-disease association prediction. *BMC bioinformatics* **20**, 1–13 (2019).
6. Bagnall, A. *et al.* Is rotation forest the best classifier for problems with continuous features? *arXiv preprint arXiv:1809.06705* (2018).
7. Pan, X. & Shen, H.-B. Scoring disease-microrna associations by integrating disease hierarchy into graph convolutional networks. *Pattern Recognit.* 107385 (2020).
8. Dong, T. N. & Khosla, M. Mucomid: A multitask convolutional learning framework for mirna-disease association prediction. *arXiv preprint arXiv:2108.04820* (2021).
9. Li, Y. *et al.* Hmdd v2. 0: a database for experimentally supported human microrna and disease associations. *Nucleic acids research* **42**, D1070–D1074 (2014).
10. Huang, Z. *et al.* Hmdd v3. 0: a database for experimentally supported human microrna–disease associations. *Nucleic acids research* **47**, D1013–D1017 (2019).

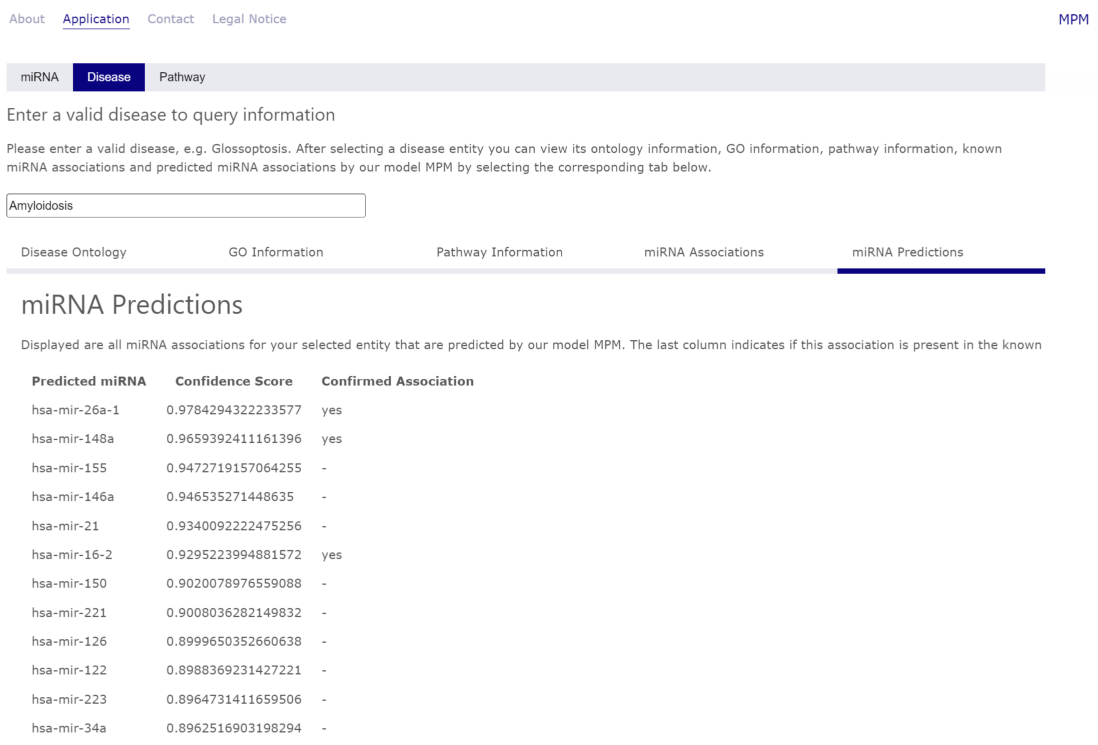

Figure 2. Web app: Display of *predicted miRNAs* for the disease *Amyloidosis*. Shows a list of all *predicted miRNA* associations (left column), with their *confidence score* (middle column) and the indication if this is a *confirmed association* (right column), in which a confirmed association is indicated with *yes* and - otherwise.

11. Bhattacharya, S., Ha-Thuc, V. & Srinivasan, P. Mesh: a window into full text for document summarization. *Bioinformatics* **27**, i120–i128 (2011).

12. Kozomara, A., Birgaoanu, M. & Griffiths-Jones, S. mirbase: from microrna sequences to function. *Nucleic acids research* **47**, D155–D162 (2019).

13. Kononenko, I. Estimating attributes: analysis and extensions of relief. In *European conference on machine learning*, 171–182 (1994).

14. Dong, T. N. & Khosla, M. Towards a consistent evaluation of mirna-disease association prediction models. In *2020 IEEE International Conference on Bioinformatics and Biomedicine (BIBM)*, 1835–1842 (IEEE, 2020).

15. Svm classifier. https://scikit-learn.org/stable/modules/generated/sklearn.svm.SVC.html.

16. Adaboost classifier. https://scikit-learn.org/stable/modules/generated/sklearn.ensemble.AdaBoostClassifier.html.

17. K-nearest neighbor classifier. https://scikit-learn.org/stable/modules/generated/sklearn.neighbors.KNeighborsClassifier.html.

18. Gaussian naive bayes classifier. https://scikit-learn.org/stable/modules/generated/sklearn.naive_bayes.GaussianNB.html.

19. Multi-layer perceptron classifier. https://scikit-learn.org/stable/modules/generated/sklearn.neural_network.MLPClassifier.html.

20. Decision tree classifier. https://scikit-learn.org/stable/modules/generated/sklearn.tree.DecisionTreeClassifier.html.

21. Fabregat, A. *et al.* The reactome pathway knowledgebase. *Nucleic acids research* **46**, D649–D655 (2018).

22. Moris, P. Python script and package for gene ontology enrichment analysis. https://pmoris.github.io/goscripts/_build/html/source/README.html.

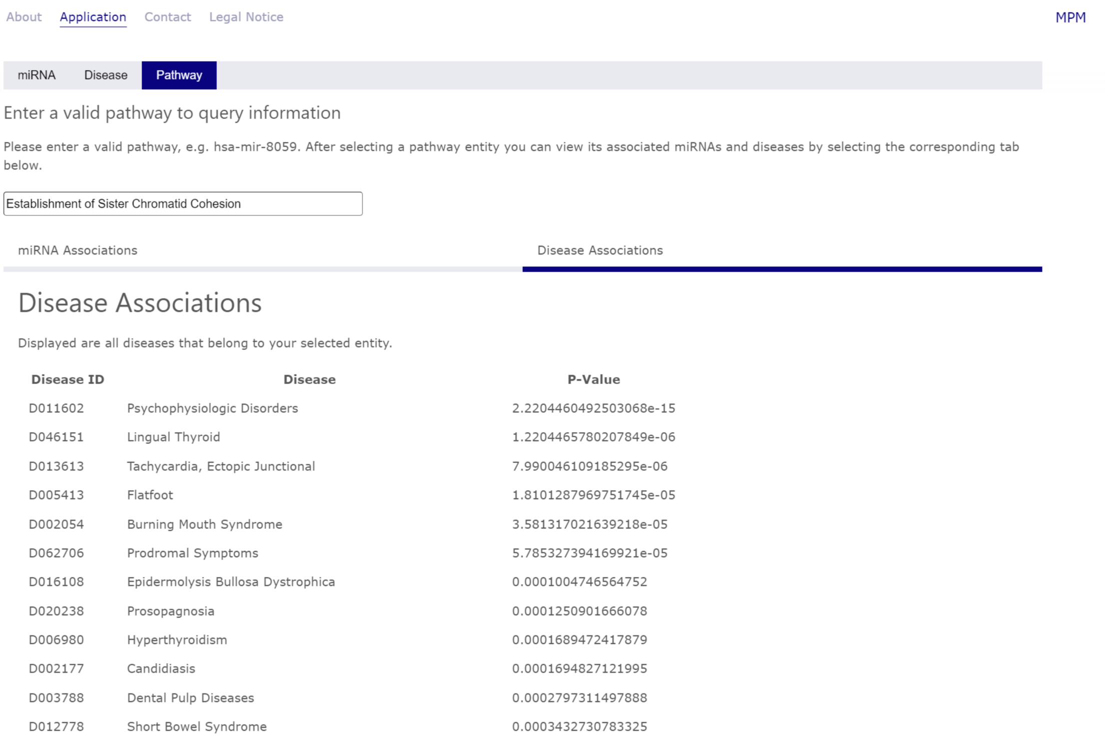

Figure 3. Web app: Display of *most significant diseases* for the pathway *Establishment of Sister Chromatid Cohesion*. The left column shows the diseases mesh ID with the corresponding disease name in the middle column. The p-value for each listed disease is displayed in the right column. The entries are ordered in ascending order by their p-value.
